# Effect of educational intervention on risk factors of cardiovascular diseases among school teachers: a quasi-experimental study in a suburb of Kolkata, West Bengal, India

**DOI:** 10.1186/s12889-023-17227-w

**Published:** 2023-11-21

**Authors:** Anubrata Karmakar, Aritra Bhattacharyya, Bijit Biswas, Aparajita Dasgupta, Lina Bandyopadhyay, Bobby Paul

**Affiliations:** 1 Department of Community Medicine, North Bengal Medical College and Hospital, Siliguri, West Bengal India; 2 Department of Community Medicine, Rampurhat Government Medical College and Hospital, Rampurhat, West Bengal India; 3 Department of Community and Family Medicine, All India Institute of Medical Sciences, Deoghar, Jharkhand India; 4 Department of Preventive and Social Medicine, All India Institute of Hygiene and Public Health, Kolkata, India

**Keywords:** Cardiovascular Disease, Health education intervention, School teachers, Risk factors, Lifestyle modifications

## Abstract

**Objective:**

This prospective study aimed to evaluate the effects of a health education intervention on cardiovascular disease (CVD) risk factors among school teachers.

**Methods:**

The study, conducted from August 2016 to May 2017, involved teachers from four schools in Baruipur, West Bengal, India. It was a multicentric, quasi-experimental study with an intervention group receiving tailored health education promoting lifestyle modifications, while the control group received no intervention. Baseline and endline assessments included behavioural and biological characteristics related to cardiovascular health and risk assessment. Data were analysed using JAMOVI.

**Results:**

The intervention group showed significant improvements in physical activity levels [Cohen’s d (Cd): 0.43, *p* = 0.006] and the consumption of fruits and vegetables (Cd: 1.00, *p* = < 0.001). Notably, there was a considerable reduction in the consumption of salt (Cd: -0.93, *p* = 0.039), oil (Cd: -0.98, *p* = < 0.001), fast food (Cd: -0.99, *p* = < 0.001), junk food (Cd: -0.99, *p* = < 0.001), and red meat (Cd: -1.00, *p* = < 0.001) among participants. However, there were no significant improvements in biological characteristics within the intervention group. In contrast, the control group exhibited no significant changes in behavioural and biological characteristics compared to baseline. The intervention group showed a minor non-significant reduction (3.0%) in their 10-year cardiovascular risk compared to baseline (Cd: -1.00, *p* = 0.50), while the control group had a negligible non-significant increase (0.7%) in their cardiovascular risk (Cd: 1.00, *p* = 1.00).

**Conclusion:**

Health education intervention positively influenced behavioural characteristics, such as physical activity and dietary habits, among school teachers. However, no significant improvements were observed in biological characteristics or cardiovascular risk factors.

**Supplementary Information:**

The online version contains supplementary material available at 10.1186/s12889-023-17227-w.

## Introduction

Cardiovascular diseases (CVDs) are a leading global cause of death, claiming over 17 million lives annually. They account for 32% of all deaths worldwide, surpassing other non-communicable diseases, cancers, respiratory diseases, and diabetes. By 2030, CVDs are projected to cause around 23.6 million deaths, with 80% occurring in low- and middle-income countries [1–4]. In India, non-communicable diseases, including CVDs, contribute to 60% of adult deaths, with CVDs alone responsible for 26% of these fatalities. Alarmingly, a significant proportion of CVD deaths occur in individuals aged 30 to 69 years, indicating an earlier onset compared to counterparts in economically developed countries. The prevalence of risk factors like sedentary lifestyles, overweight/obesity, and hypertension further exacerbates the situation [5–7].

Cardiovascular diseases (CVDs) encompass conditions affecting the heart, blood vessels, and brain, including heart attacks, strokes, and arterial diseases. CVD risk factors can be modifiable (e.g., tobacco use, physical inactivity, unhealthy diet, high blood pressure, obesity) or non-modifiable (e.g., age, gender, genetics). Lifestyle modifications are crucial for preventing and managing CVDs, and lifestyle programs involving healthy habits, dietary counselling, exercise training, and behavioural changes have proven effective in reducing heart disease, stroke, and diabetes. By addressing obesity, promoting healthy diets, and increasing physical activity, up to 80% of these diseases can be prevented. It is important to develop impactful strategies for screening, prevention, and intervention to combat CVDs [8–10].

Teaching, a sedentary occupation, increases the risk of non-communicable diseases, including cardiovascular diseases (CVDs) [11]. Limited research exists on CVD risk factors among teachers, despite their vulnerability to such diseases [12]. This study in Baruipur, India, aims to assess the impact of health education interventions on modifying CVD risk factors among school teachers. By improving knowledge and reducing risk factors, the study seeks to enhance intervention programs for teachers’ cardiovascular health, yielding positive outcomes. These findings can inspire policymakers to implement similar interventions for teachers and students, reducing the burden of non-communicable diseases, especially CVDs. With CVDs remaining a significant global health challenge, India has witnessed a rise in CVD-related deaths in urban and rural areas. Targeting modifiable risk factors becomes crucial for preventing and managing CVDs. Leveraging the influential role of school teachers provides a unique opportunity for interventions promoting healthier behaviours and reducing CVD risk factors.

## Methods

### Participants

This multicentric quasi-experimental study took place from August 2016 to May 2017 among teachers in four schools in Baruipur, a Kolkata suburb, West Bengal, India. We used simple random sampling (SRS) with replacement to select four clusters out of 18 in the Baruipur block, each containing an average of 18.5 ~ 19 schools. From each cluster, one school was chosen [13].

After securing permission and informed written consent from school heads, all teachers working in the study schools during baseline assessments were included. The four study schools were then randomly assigned to either the intervention or control group. Sample sizes were determined based on parameters from a similar study by Awosan et al. [12] in Nigeria since there were no prior Indian studies available. For systolic blood pressure (SBP), we estimated a mean difference of 2.87 with a standard deviation of 9.2, requiring a sample size of 66 per arm for 80% power and a 5% significance level. Similarly, for fasting blood sugar (FBS) and total blood cholesterol, sample sizes of 17 in each arm were calculated to detect differences of 6.69 mg/dl and 12.71 mg/dl, respectively, with their respective standard deviations [12]. We used the online sample size calculator Statulator [14] for these calculations.

The study enrolled 68 teachers in the intervention group and 62 in the control group, totalling 130 participants. Data collection schedules were coordinated with school authorities. We utilized a pre-designed, pretested, self-administered questionnaire, clinical examination, and laboratory blood investigations. Blood pressure and anthropometric measurements adhered to standard operating procedures. Female teachers received examinations with a female attendant to ensure privacy. Teachers arrived at school one hour before classes, following a 12-hour fasting period, for blood sample collection.

### Intervention

We created an educational module covering key aspects of cardiovascular diseases (CVDs): common types, modifiable and non-modifiable risk factors, prevention, diabetes and obesity complications, and guidance on healthy diet, physical activity, stress management, and quitting addictions. This module adhered to guidelines from the National Institute of Nutrition, Indian Council of Medical Research (ICMR), and the World Health Organization (WHO) [15, 16]. We also prepared PowerPoint presentations (Annexure I) to enhance interactive lectures.

**Fig. 1:**
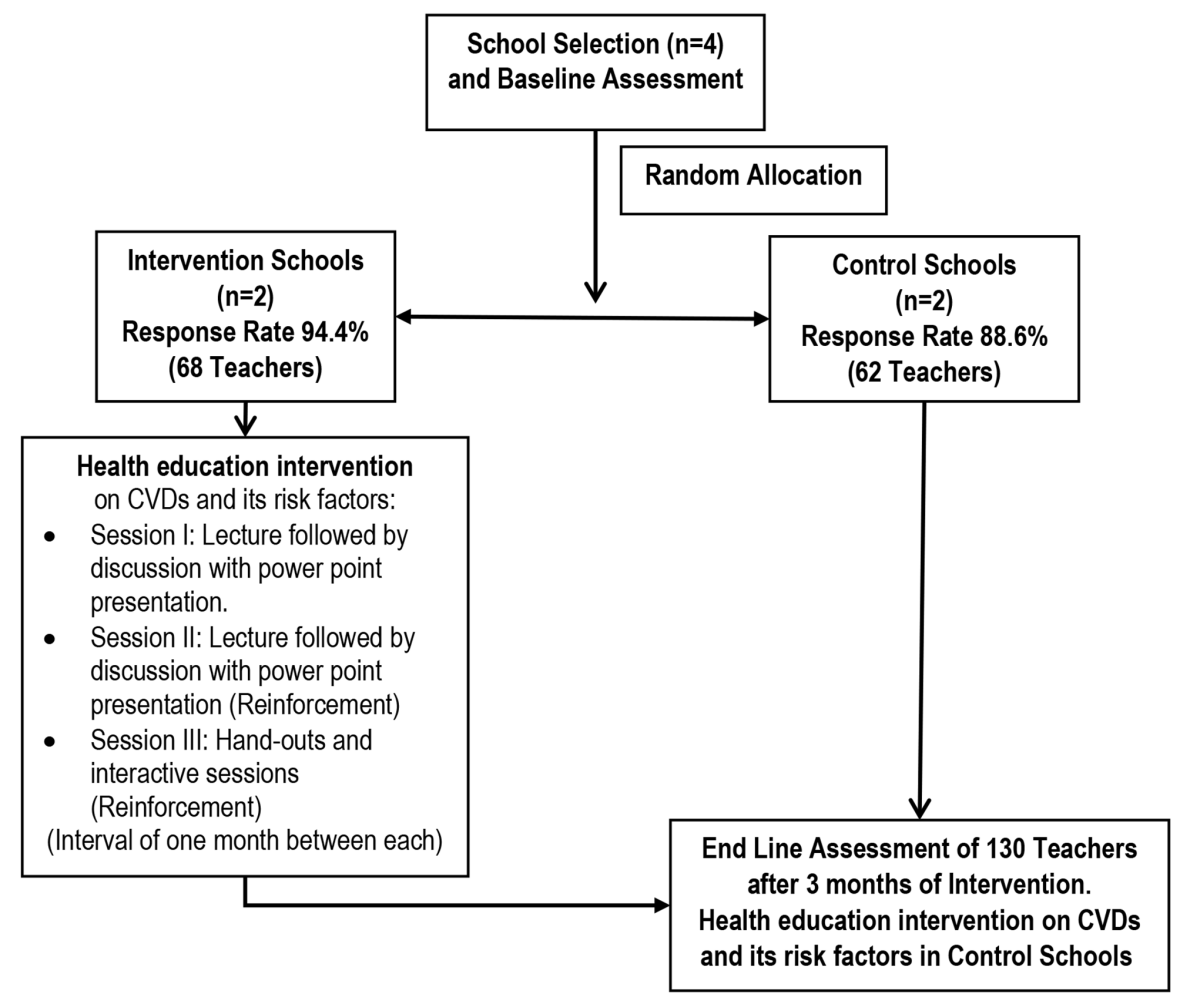
Flowchart indicating the study process

We conducted intervention sessions, approved by the Teacher-in-charge, in the teachers’ room or school auditorium using a laptop and projector, held on Saturdays after school hours without disrupting regular academic activities. We reinforced learning by repeating lectures twice per school with a one-month gap between. Interactive sessions addressed questions and clarified content. Handouts (Annexure II) aided retention. Three months post-intervention, we conducted an endline assessment using the same tools as the baseline, excluding background characteristics. Control schools received subsequent intervention sessions. Figure 1 illustrates the study processes.

### Measures

#### Behavioural characteristics

Physical activity levels were assessed using the International Physical Activity Questionnaire Short Form (IPAQ-SF), with individuals reporting ≥ 600 metabolic equivalent (MET) per week classified as physically active [17, 18]. Meeting the recommended salt intake of < 5 g per day and consuming < 500 ml of oil per month aligned with guidelines [16, 19]. Meeting the recommendation for fruit and vegetable consumption meant consuming ≥ 400 g daily [19]. Tobacco users were defined as those reporting tobacco use in the past 30 days [20]. Stress levels were measured using the Perceived Stress Scale (PSS), consisting of four items, with higher scores indicating increased stress levels [21].

#### Biological characteristics

We adhered to standard operating procedures for measuring blood pressure, height, weight, waist circumference, and hip circumference. Fasting blood sugar (FBS) and lipid profiles, including total cholesterol, low-density lipoprotein (LDL), high-density lipoprotein (HDL), and triglycerides, were assessed using established protocols [19, 20]. Individuals taking antihypertensive medication or with systolic blood pressure (SBP) ≥ 140 mm Hg and/or diastolic blood pressure (DBP) ≥ 90 mm Hg were classified as hypertensive. Those on antidiabetic medication or with FBS ≥ 126 mg/dl were considered diabetic. Obesity was defined as a body mass index (BMI) ≥ 25 kg/m², and central obesity as waist circumference > 80 cm for females and > 90 cm for males. A waist-hip ratio > 0.85 for females and > 90 cm for males indicated a higher risk.

#### Cardiovascular risk scores

We identified a higher risk for cardiovascular disease (CVD) as total cholesterol levels ≥ 200 mg/dl, LDL ≥ 130 mg/dl, and triglycerides ≥ 150 mg/dl, while a cardioprotective HDL level was defined as < 50 mg/dl for women and < 40 mg/dl for men [15, 19, 20, 22]. We calculated the Framingham 10-year cardiovascular risk score using age, sex, total and HDL cholesterol, smoking status, and blood pressure, following established calculation guidelines [23, 24]. The prevalence of metabolic syndrome was determined according to the new International Diabetes Federation (IDF) definitions [25].

### Statistical analysis plan

We conducted data analysis using JAMOVI (version 2.3.26), an open-source statistical software [26]. Initially, we inputted the data into Microsoft Excel and then imported it into JAMOVI for analysis. Descriptive statistics included reporting quantitative and qualitative variables, using frequency (percentage) and median [interquartile range (IQR)]. To compare baseline and endline behavioural and biological characteristics related to cardiovascular health between the intervention and control groups, we employed the Mann-Whitney U test for qualitative variables and the Chi-square test for quantitative variables. Within each group, we used the Wilcoxon matched pair signed rank test for qualitative variables and McNemar’s test for quantitative variables to compare baseline and endline characteristics. We measured the effect size using Cohen’s D. In all quantitative analyses, a *p*-value < 0.05 was considered statistically significant, indicating significant differences between groups or within groups over time.

## Results

In the intervention group, the median age of teachers was 36.5 years (IQR: 11), while in the control group, it was 37 years (IQR: 9). The median per capita monthly family income (PCMI) for the intervention and control groups was 143.2 USD (IQR: 116.2) and 147.7 USD (IQR: 120.5) respectively. Furthermore, the median years of experience for teachers in the intervention and control groups were 121.5 months (IQR: 116) and 107.7 months (IQR: 72) respectively. As for the median number of classes taught by them, it was 25 (IQR: 6) for the intervention group and 27 (IQR: 6) for the control group. It is important to note that there was a statistically significant difference in terms of background characteristics between the intervention and control groups. (Table 1)

**Table 1:** Distribution of the study participants according to their background characteristics (n = 130)

Variables	Intervention Group (n = 68)	Control Group (n = 62)	Total (n = 130)	*p*-value ^a^
	N (%)	N (%)	N (%)	
**Age (in completed years)**				
24–36	34 (50.0)	27 (43.5)	61 (46.9)	0.157
37–48	23 (33.8)	30 (48.4)	53 (40.8)	
49–60	11 (16.2)	5 (8.1)	16(12.3)	
**Sex**				
Male	46 (67.6)	32 (51.6)	78 (60)	0.062
Female	22 (32.4)	30 (48.4)	52 (40)	
**Religion**				
Hindu	62 (91.2)	59 (95.2)	121 (93.1)	0.497
Muslim	6 (8.8)	3 (4.8)	9 (6.9)	
**Marital Status**				
Currently married	59 (86.8)	51 (82.3)	110 (84.6)	0.447
Others	9 (13.2)	11 (17.7)	20 (15.4)	
**Level of education**				
Graduate	19 (27.9)	16 (25.8)	35 (26.9)	0.784
Post-graduate	49 (72.1)	46 (74.2)	95 (73.1)	
**Type of Family**				
Nuclear	33 (48.5)	25 (40.3)	58 (44.6)	0.347
Joint	35 (51.5)	37 (59.7)	72 (55.4)	
**Place of stay**				
Rural	26 (38.2)	28 (45.2)	54 (41.5)	0.423
Urban	42 (61.8)	34 (54.8)	76 (58.5)	
**Education level of spouse/father**				
Up to Higher secondary	8 (11.8)	7 (11.3)	15 (11.5)	0.142
Graduate	33 (48.5)	40 (64.5)	73 (56.2)	
Post-graduate	27 (39.7)	15 (24.2)	42 (32.3)	
**Occupation of spouse/father**				
Homemaker	37 (54.4)	21 (33.9)	58 (44.6)	0.089
Teacher	12 (17.6)	9 (14.5)	21 (16.2)	
Service	9 (13.2)	17 (27.4)	26 (20)	
Business	5 (7.4)	7 (11.3)	12 (9.2)	
Others	5 (7.4)	8 (12.9)	13 (10)	
**PCMI**: (in USD)				
Q1 (24.2–91.7)	18 (26.5)	14 (22.6)	32 (24.6)	0.963
Q2 (91.8-145.2)	16 (23.5)	15 (24.2)	31 (23.8)	
Q3 (145.3-209.5)	18 (26.5)	17 (27.4)	35 (26.9)	
Q4 (209.6–1291)	16 (23.5)	16 (25.8)	32 (24.6)	
**Subject of specialization**				
Language ^b^	23 (33.8)	15 (24.2)	38 (29.2)	0.891
Mathematics	7 (10.3)	6 (9.7)	13 (10)	
Science ^c^	13 (19.1)	15 (24.2)	28 (21.5)	
Social studies ^d^	13 (19.1)	13 (21.0)	26 (20)	
Other science subjects ^e^	3 (4.4)	3 (4.8)	6 (4.6)	
Other arts or commerce subjects ^f^	9 (13.2)	10 (16.1)	19 (14.6)	
**Duration in teaching profession in completed months**				
1-100	28 (41.2)	28 (45.2)	56 (43.1)	0.391
101–200	25 (36.8)	26 (41.9)	51 (39.2)	
≥ 201	15 (22.1)	8 (12.9)	23 (17.7)	
**Average number of classes taken/ week**				
6–15	5 (7.4)	2 (3.2)	7 (5.4)	0.351
16–25	33 (48.5)	26 (41.9)	59 (45.4)	
26–35	30 (44.1)	34 (54.8)	64 (49.2)	
**Cardiovascular comorbidities**:				
Ischemic heart disease	3 (4.4)	2 (3.2)	5 (3.8)	0.725
Hypertension	9 (13.2)	7 (11.3)	16 (12.3)	0.736
Diabetes	6 (8.8)	3 (4.8)	9 (6.9)	0.371

^a^ Chi square test; ^b^ Bengali, English; ^c^ Physics, Chemistry, Biological Science; ^d^ History, Geography, ^e^ Environmental science, Computer science, Nutrition; ^f^ Education, political science, Economics, Physical education, Sanskrit, Accountancy; PCMI: per capita monthly family income, Q: quartile

The intervention group demonstrated significant improvements in physical activity levels (effect size: 0.43, *p* = 0.006) and increased consumption of fruits and vegetables (effect size: 1.00, *p* = < 0.001). Importantly, there was a notable decrease in the consumption of salt (effect size: -0.93, *p* = 0.039), oil (effect size: -0.98, *p* = < 0.001), fast food (effect size: -0.99, *p* = < 0.001), junk food (effect size: -0.99, *p* = < 0.001), and red meat (effect size: -1.00, *p* = < 0.001) among participants. However, when it came to biological characteristics, no significant improvements were noted in these parameters. In contrast, the control group did not show any significant changes in terms of behavioural and biological characteristics compared to the baseline assessments. Significant differences were observed between the intervention and control groups at baseline and at the end in terms of total cholesterol levels. Additionally, there were significant differences between the groups in baseline and endline measurements of FBS, WC, BMI, and triglycerides. (Tables 2 and 3)

**Table 2:** Comparison of teachers in the intervention and control schools according to their various behavioral characteristics related to cardiovascular heath at baseline and end of the study (n = 130)

Characteristics	Intervention Group (n = 68) Median (IQR) / N [%}	Control Group (n = 62) Median (IQR) / N [%}	*p*-value	Effect Size
**Physical activity (MET-min/week)**				
Before the intervention	636.0 (618.0)	586.5 (541.0)	0.618^a^	0.05^b^
After the intervention	855.0 (414.0)	603.5 (425.0)	**< 0.001** ^**a**^	0.47^b^
*p*-value	**0.006** ^**c**^	0.082^c^		
Effect Size	0.43^b^	-0.42^b^		
**Physical activity status**: (Active) ^f^				
Before the intervention	37 [54.4]	30 [48.4]	0.492^e^	-
After the intervention	61 [89.7]	31 [50.0]	**< 0.001** ^**e**^	
*p*-value	**< 0.001** ^**d**^	1.00^d^		
**Salt consumption (grams/day)**				
Before the intervention	8.3 (2.6)	8.3 (2.5)	0.850^a^	0.02^b^
After the intervention	5.6 (1.7)	7.3 (1.7)	**< 0.001** ^**a**^	-0.58^b^
*p*-value	**< 0.001** ^**c**^	0.131^c^		
Effect Size	-0.93^b^	-0.37^b^		
**Salt consumption level**: (As recommended) ^g^				
Before the intervention	9 [13.2]	6 [9.7]	0.526^e^	-
After the intervention	16 [23.5]	5 [8.1]	**0.017** ^**e**^	
*p*-value	**0.039** ^**d**^	1.00^d^		
**Oil consumption (ml/month)**				
Before the intervention	1.0 (0.3)	0.9 (0.2)	0.851^a^	0.02^b^
After the intervention	0.8 (0.2)	1.0 (0.3)	**< 0.001** ^**a**^	-0.48^b^
*p*-value	**< 0.001** ^**c**^	0.109^c^		
Effect Size	-0.98^b^	0.27^b^		
**Oil consumption level**: (As recommended) ^h^				
Before the intervention	1 [1.5]	0 [0.0]	0.338^e^	-
After the intervention	0 [0.0]	0 [0.0]	-	
*p*-value	1.00^d^	1.00^d^		
**Fruits and vegetable consumption (grams/day)**				
Before the intervention	157.1 (125.0)	150.0 (114.3)	0.551^a^	0.06^b^
After the intervention	285.7 (135.7)	157.1 (100.0)	**< 0.001** ^**a**^	0.78^b^
*p*-value	**< 0.001** ^**c**^	0.959^c^		
Effect Size	1.00^b^	-0.01^b^		
**Fruits and vegetable consumption level**: (As recommended) ^i^				
Before the intervention	2 [2.9]	4 [6.5]	0.341^e^	-
After the intervention	14 [20.6]	5 [8.1]	**0.043** ^**e**^	
*p*-value	**< 0.001** ^**d**^	1.00^d^		
**Junk food consumption (times/week)**				
Before the intervention	2.0 (4.0)	2.0 (4.0)	0.513^a^	0.06^b^
After the intervention	0.0 (1.0)	2.0 (4.0)	**< 0.001** ^**a**^	-0.51^b^
*p*-value	**< 0.001** ^**c**^	0.134^c^		
Effect Size	-0.99^b^	-0.34^b^		
**Fast foods consumption (times/week)**				
Before the intervention	4.0 (8.0)	4.0 (6.0)	0.366^a^	0.09^b^
After the intervention	0.5 (2.0)	4.0 (6.0)	**< 0.001** ^**a**^	0.56^b^
*p*-value	**< 0.001** ^**c**^	0.201^c^		
Effect Size	-0.99^b^	-0.25^b^		
**Red meat consumption (times/month)**				
Before the intervention	1.0 (2.0)	1.0 (2.0)	0.973^a^	0.00^b^
After the intervention	0.0 (1.0)	1.0 (2.0)	**< 0.001** ^**a**^	-0.37^b^
*p*-value	**< 0.001** ^**c**^	0.934^c^		
Effect Size	-1.00^b^	0.03^b^		
**Tobacco User**: (Yes)				
Before the intervention	12 [17.6]	7 [11.3]	0.305^e^	-
After the intervention	11 [16.2]	7 [11.3]	0.420^e^	
*p*-value	1.00^d^	1.00^d^		
**Smoker**: (Yes)				
Before the intervention	11 [16.2]	5 [8.1]	0.160^e^	-
After the intervention	10 [14.7]	5 [8.1]	0.236^e^	
*p*-value	1.00^d^	1.00^d^		

^a^ Mann-Whitney U test; ^b^ Cohen’s D, ^c^ Wilcoxon matched pair signed rank test; ^d^ McNemar’s test; ^e^ Chi square test; ^f^ physical activity ≥ 600 MET-min/ week; ^g^ Salt consumption < 5gm/day; ^h^ Oil consumption < 500 ml / month; ^i^ Fruit and vegetable consumption ≥ 400 gm / day; IQR: interquartile range; MET: metabolic equivalent

**Table 3:** Comparison of teachers in the intervention and control schools according to their various biological characteristics related to cardiovascular heath at baseline and end of the study (n = 130)

Characteristics	Intervention Group (n = 68) Median (IQR) / N [%}	Control Group (n = 62) Median (IQR) / N [%}	*p*-value	Effect Size
**Perceived Stress score**				
Before the intervention	5.5 (5.0)	5.0 (5.0)	0.856^a^	0.02^b^
After the intervention	6.0 (3.0)	5.0 (4.0)	0.968^a^	0.00^b^
*p*-value	0.882^c^	0.845^c^		
Effect Size	-0.03^b^	-0.03^b^		
**Systolic Blood pressure (mm of Hg)**				
Before the intervention	125 (23.0)	121.5 (16.0)	0.330^a^	0.09^b^
After the intervention	124 (23.0)	121.5 (17.0)	0.374^a^	0.09^b^
*p*-value	0.527^c^	0.887^c^		
Effect Size	-0.28^b^	0.05^b^		
**Diastolic Blood pressure (mm of Hg)**				
Before the intervention	81.5 (12.5)	82.0 (10.0)	0.902^a^	0.01^b^
After the intervention	80.5 (12.3)	82.0 (10.0)	0.915^a^	0.01^b^
*p*-value	0.062^c^	0.592^c^		
Effect Size	-0.82^b^	0.22^b^		
**Blood pressure**: (High)^f^				
Before the intervention	20 [29.4]	11 [17.7]	0.119^e^	-
After the intervention	17 [25.0]	8 [12.9]	0.080^e^	
*p*-value	0.250^d^	0.250^d^		
**FBS (mg/dl)**				
Before the intervention	94.5 (18.0)	87.0 (11.0)	**< 0.001** ^**a**^	0.34^b^
After the intervention	95.0 (17.3)	86.0 (11.0)	**< 0.001** ^**a**^	0.36^b^
*p*-value	0.294^c^	0.842^c^		
Effect Size	-0.44^b^	-0.07^b^		
**FBS**: (High)^g^				
Before the intervention	6 [8.8]	2 [3.2]	0.185^e^	-
After the intervention	3 [4.4]	2 [3.2]	0.725^e^	
*p*-value	0.250^d^	1.00^d^		
**BMI (kg/m** ^**2)**^				
Before the intervention	25.4 (3.8)	23.7 (4.9)	**0.011** ^**a**^	0.26^b^
After the intervention	25.4 (4.0)	23.7 (5.1)	**0.013** ^**a**^	0.25^b^
*p*-value	0.181^c^	0.709^c^		
Effect Size	-1.00^b^	0.10^b^		
**Obesity**: (Yes)^h^				
Before the intervention	36 [52.9]	23 [37.1]	0.070^e^	-
After the intervention	36 [52.9]	22 [35.5]	**0.046** ^**e**^	
*p*-value	1.00^d^	1.00^d^		
**Waist circumference (cm)**				
Before the intervention	90.0 (10.0)	86.5 (13.0)	**0.002** ^**a**^	0.31^b^
After the intervention	90.0 (10.0)	86.5 (13.0)	**0.002** ^**a**^	0.31^b^
*p*-value	0.346^c^	-		
Effect Size	-1.00^b^	-		
**Central obesity**: (Yes)^i^				
Before the intervention	42 [61.8]	34 [54.8]	0.423^e^	-
After the intervention	42 [61.8]	34 [54.8]	0.423^e^	
*p*-value	1.00^d^	1.00^d^		
**Waist-hip ratio**: (High)^j^				
Before the intervention	57 [83.8]	49 [79.0]	0.482^e^	-
After the intervention	57 [83.8]	49 [79.0]	0.482^e^	
*p*-value	1.00^d^	1.00^d^		
**Total cholesterol (mg/dl)**				
Before the intervention	177 (56)	167.0 (36.0)	**0.043** ^**a**^	0.21^b^
After the intervention	175 (56)	166.2 (35.0)	0.080^a^	0.18^b^
*p*-value	0.050	0.790		
Effect Size	-0.86^b^	-0.11^b^		
**Total cholesterol**: (High)^k^				
Before the intervention	22 [32.4]	10 [16.1]	**0.032** ^**e**^	
After the intervention	22 [32.4]	10 [16.1]	**0.032** ^**e**^	
*p*-value	1.00^d^	1.00^d^		
**LDL (mg/dl)**				
Before the intervention	89.5 (50.0)	84.0 (29.0)	0.348^a^	0.09^b^
After the intervention	86.0 (50.0)	85.5 (32.0)	0.493^a^	0.07^b^
*p*-value	0.050^c^	0.255^c^		
Effect Size	-0.81^b^	0.32^b^		
**LDL**: (High)^l^				
Before the intervention	11 [16.2]	8 [12.9]	0.598^e^	-
After the intervention	11 [16.2]	7 [11.3]	0.420^e^	
*p*-value	1.00^d^	1.00^d^		
**Triglyceride (mg/dl)**				
Before the intervention	141.5 (83.0)	126.5 (21.0)	**0.017** ^**a**^	0.24^b^
After the intervention	140.5 (83.0)	127.5 (53.0)	**0.030** ^**a**^	0.22^b^
*p*-value	0.483^c^	0.721^c^		
Effect Size	-0.31^b^	0.07^b^		
**Triglyceride**: (High)^m^				
Before the intervention	31 [45.6]	20 [32.3]	0.120^e^	-
After the intervention	30 [44.1]	20 [32.3]	0.165^e^	
*p*-value	1.00^d^	1.00^d^		
**HDL (mg/dl)**				
Before the intervention	55.0 (16.3)	53.0 (8.0)	0.436^a^	0.07^b^
After the intervention	55.0 (16.3)	53.0 (8.0)	0.268^a^	0.11^b^
*p*-value	0.079^c^	0.476^c^		
Effect Size	0.72^b^	-0.22^b^		
**HDL**: (Low)n				
Before the intervention	59 [86.8]	57 [91.9]	0.342^e^	-
After the intervention	60 [88.2]	54 [87.1]	0.844^e^	
*p*-value	1.00^d^	0.250^d^		
**Dyslipidemia**: (Yes)				
Before the intervention	37 [54.4]	27 [43.5]	0.216^e^	-
After the intervention	36 [52.9]	27 [43.5]	0.284^e^	
*p*-value	1.00^d^	1.00^d^		
**Metabolic syndrome**: (Yes)				
Before the intervention	15 [22.1]	6 [9.7]	0.055^e^	-
After the intervention	14 [20.6]	8 [12.9]	0.243^e^	
*p*-value	1.00^d^	0.500^d^		

^a^ Mann-Whitney U test; ^b^ Cohen’s D, ^c^ Wilcoxon matched pair signed rank test; ^d^ McNemar’s test; ^e^ Chi square test; ^f^ SB*P* ≥ 140 mm Hg or DB*P* ≥ 90 mm Hg or both; ^g^ FBS ≥ 126 mg/dl; ^h^ BMI ≥ 25 kg / m^2^; ^i^ Waist circumference > 90 for males and > 80 for females; ^j^ Waist-hip ratio > 0.90 for males > 0.85 for females; ^k^ total cholesterol ≥ 200 mg /dl, ^l^ LDL ≥ 130 mg / dl, ^m^ triglycerides ≥ 150 mg / dl; ^n^ HDL < 40 mg/dl for males and < 50 mg/dl for females. IQR: interquartile range; SBP: systolic blood pressure; DBP: diastolic blood pressure; FBS: fasting blood sugar; LDL: low density lipoprotein; HDL: high density lipoprotein

There was a significant difference (effect size: 0.19, *p* = 0.035) in the 10-year cardiovascular risk between the intervention and control groups at baseline, with teachers in the intervention group having a higher risk. However, this difference became non-significant post-intervention (effect size: 0.14, *p* = 0.106). The intervention group exhibited a small non-significant decrease (3.0%) in their 10-year cardiovascular risk compared to baseline (effect size: -1.00, *p* = 0.50). In contrast, the control group showed a slight non-significant increase (0.7%) in their cardiovascular risk compared to baseline (effect size: 1.00, *p* = 1.00). (Table 4)

**Table 4:** Comparison of teachers in the intervention and control schools according to their cardiovascular risk at baseline and end of the study (n = 130)

Characteristics	Intervention Group (n = 68) Median (IQR) / N [%}	Control Group (n = 62) Median (IQR) / N [%}	*p*-value	Effect Size
**Framingham Cardiovascular Risk Score:**				
Before the intervention	0.0 (2.0)	0.0 (1.0)	**0.035** ^**a**^	0.19^b^
After the intervention	0.0 (2.0)	0.0 (1.0)	0.106^a^	0.14^b^
*p*-value	0.174^c^	0.181^c^		
Effect Size	-1.00^b^	1.00^b^		
**10-year Cardiovascular Risk**: (≥ 1%)				
Before the intervention	31 [45.6]	19 [30.6]	0.080^e^	-
After the intervention	29 [42.6]	20 [32.3]	0.222^e^	
*p*-value	0.50^d^	1.00^d^		

^a^ Mann-Whitney U test; ^b^ Cohen’s D, ^c^ Wilcoxon matched pair signed rank test; ^d^ McNemar’s test; ^e^ Chi square test; IQR: interquartile range

## Discussion

The study aimed to assess the impact of an educational intervention program on cardiovascular disease (CVD) risk factors among school teachers, focusing on their background, behavioral, biological characteristics, and cardiovascular risk.

The intervention group displayed notable improvements in physical activity levels and increased consumption of fruits and vegetables, indicating the program’s effectiveness in promoting positive behavioral changes. These findings are consistent with prior research highlighting the success of health education interventions in encouraging physical activity and healthier dietary habits [27–32]. Moreover, the intervention group significantly reduced their consumption of salt, oil, fast food, junk food, and red meat, aligning with established recommendations for cardiovascular health [33, 34].

Despite these favourable behavioural changes, the study did not observe significant improvements in assessed biological characteristics. This suggests that while the intervention positively impacted behaviour, it did not produce notable physiological changes within the study’s timeframe. Similar observations in other studies emphasize the complexity of modifying biological markers through behavioural interventions alone [35, 36]. Meta-analysis findings also indicate that lifestyle modifications may require over twelve months to effectively reduce blood pressure, particularly among Asian populations [37]. Our study had a shorter post-intervention follow-up of six months, potentially insufficient to produce significant blood pressure changes compared to baseline. In contrast, a prior intervention study by Awosan et al. [12] in Nigeria reported significant reductions in blood pressure, fasting blood sugar (FBS), and total blood cholesterol at three months post-intervention. The differences could stem from Awosan et al.‘s [12] additional incorporation of exercise and dietary control in their intervention compared to our solely communication-focused approach. Moreover, the frequency of health communication sessions differed, with Awosan et al. [12] conducting fortnightly sessions for three months, while our study featured monthly sessions over the same duration.

Interestingly, the intervention group had a higher 10-year cardiovascular risk than the control group at baseline, which became non-significant post-intervention. Although the decrease in cardiovascular risk within the intervention group was not statistically significant, it indicated a positive trend. This aligns with previous studies demonstrating reductions in cardiovascular risk through lifestyle modifications, albeit potentially requiring longer-term follow-up to detect significant changes [12, 38]. Additionally, significant baseline differences between the intervention and control groups in total cholesterol, triglycerides, fasting blood sugar (FBS), waist circumference (WC), and body mass index (BMI) were observed. Most of these differences persisted at the end of the study, possibly explaining the absence of significant cardiovascular risk differences at the endline assessments across groups.

In future research, it is recommended to conduct randomized controlled trials (RCTs) instead of quasi-experimental designs to minimize bias and confounding variables, thus providing stronger evidence for the effectiveness of interventions. Furthermore, expanding the geographical scope of the study to include diverse populations with different socioeconomic backgrounds is crucial to enhance the generalizability of the findings. Moreover, longer intervention and follow-up periods should be implemented to assess the sustained effects of interventions and observe significant changes in biological characteristics. Additionally, it is suggested that future interventions should incorporate exercise schedules and dietary control alongside health communication to enhance their effectiveness in achieving desired outcomes. Lastly, the use of objective measurements, such as objective physical activity monitoring, dietary practices monitoring, and biomarkers, would greatly enhance the validity and reliability of the collected data. These recommendations will contribute to advancing knowledge in the field and improving the quality of cardiovascular health interventions.

The study had several limitations. Firstly, it adopted a quasi-experimental design instead of a randomized controlled trial, which introduces potential bias and confounding variables. This was due to limited resources caused by a lack of funding. Secondly, the study was conducted in a limited geographical area, specifically four schools located in Baruipur, a suburb on the outskirts of Kolkata, West Bengal, India. Therefore, caution should be exercised when generalizing the findings to other geographical locations or populations with different socioeconomic backgrounds. Thirdly, the intervention and follow-up period were relatively short, which might have restricted the ability to observe significant changes in certain biological characteristics, such as blood pressure. Fourthly, unlike some previous studies that incorporated exercise schedules and dietary control in addition to health communication, our intervention solely focused on health communication. The absence of dietary and exercise control elements may have influenced the effectiveness of the intervention in achieving specific outcomes. Lastly, some of the data collected, including physical activity levels and dietary habits, relied on self-reported information, which is susceptible to recall bias or social desirability bias. These potential biases could have impacted the accuracy of the results.

In summary, the intervention program yielded positive results in terms of behavioural characteristics, such as increased physical activity and healthier dietary habits, among school teachers. However, no significant improvements were noted in biological characteristics and cardiovascular risk factors. These findings underscore the necessity for further fine-tuning and tailoring of the intervention program to effectively target the physiological aspects associated with cardiovascular disease (CVD) risk. It is imperative that health professionals, educators, and policymakers collaborate to develop precise interventions that comprehensively address CVD risk factors and promote optimal cardiovascular health among school teachers.

### Electronic supplementary material

Below is the link to the electronic supplementary material.

Annexure I: Cardiovascular Diseases: Risk factors and Prevention

Annexure II: Handout Given to Partcipants along with Intervention

## Data Availability

The datasets utilized and analysed in the present study can be obtained from the corresponding author upon a reasonable request.
