# Supplementary material for: Effect of educational intervention on risk factors of cardiovascular diseases among school teachers: a quasi-experimental study in a suburb of Kolkata, West Bengal, India
Source: BMC Public Health. 2023 Nov 21;23:2304. doi: 10.1186/s12889-023-17227-w (PMC10664257; doi:10.1186/s12889-023-17227-w)
Supplement: Supplementary file 1 — Annexure I: Cardiovascular Diseases: Risk factors and Prevention [file 12889_2023_17227_MOESM1_ESM.pdf]

# Cardiovascular Diseases: Risk factors and Prevention

Presented by-
Dr. Anubrata Karmakar
MD CM PGT
All India Institute of Hygiene & Public Health,
Kolkata

# What are cardiovascular diseases (CVDs)?

| CVDs due to atherosclerosis | Other cardiovascular diseases |
|---|---|
| 1. Ischaemic heart disease or coronary artery disease (e.g. heart attack)<br>2. Cerebrovascular disease (e.g. stroke)<br>3. Diseases of the aorta and arteries, including hypertension and peripheral vascular disease. | 1. Congenital heart disease<br>2. Rheumatic heart disease<br>3. Cardiomyopathies<br>4. Cardiac arrhythmias |

# What are the Risk Factors?

| Behavioural risk factors | Metabolic risk factors | Other risk factors |
|---|---|---|
| 1. tobacco use<br>2. physical inactivity<br>3. unhealthy diet (rich in salt, fat and calories)<br>4. harmful use of alcohol. | 5. raised blood pressure (hypertension)<br>6. raised blood sugar (diabetes)<br>7. raised blood lipids (e.g. cholesterol)<br>8. overweight and obesity. | 9. poverty and low educational status<br>10. advancing age<br>11. gender<br>12. inherited (genetic) disposition (**Family History**)<br>13. psychological factors (e.g. stress, depression) |
| **Modifiable** | | |

# Prevention: reducing exposure to risk factors

- To prevent heart attacks and strokes, the total cardiovascular risk needs to be reduced by lowering all modifiable risk factors.

- Cardiovascular risk factors such as hypertension, diabetes and hyperlipidaemia cluster together and are major risk factors for strokes and heart attacks

- CVD Prevention needs a life course approach

# DIETARY HABITS

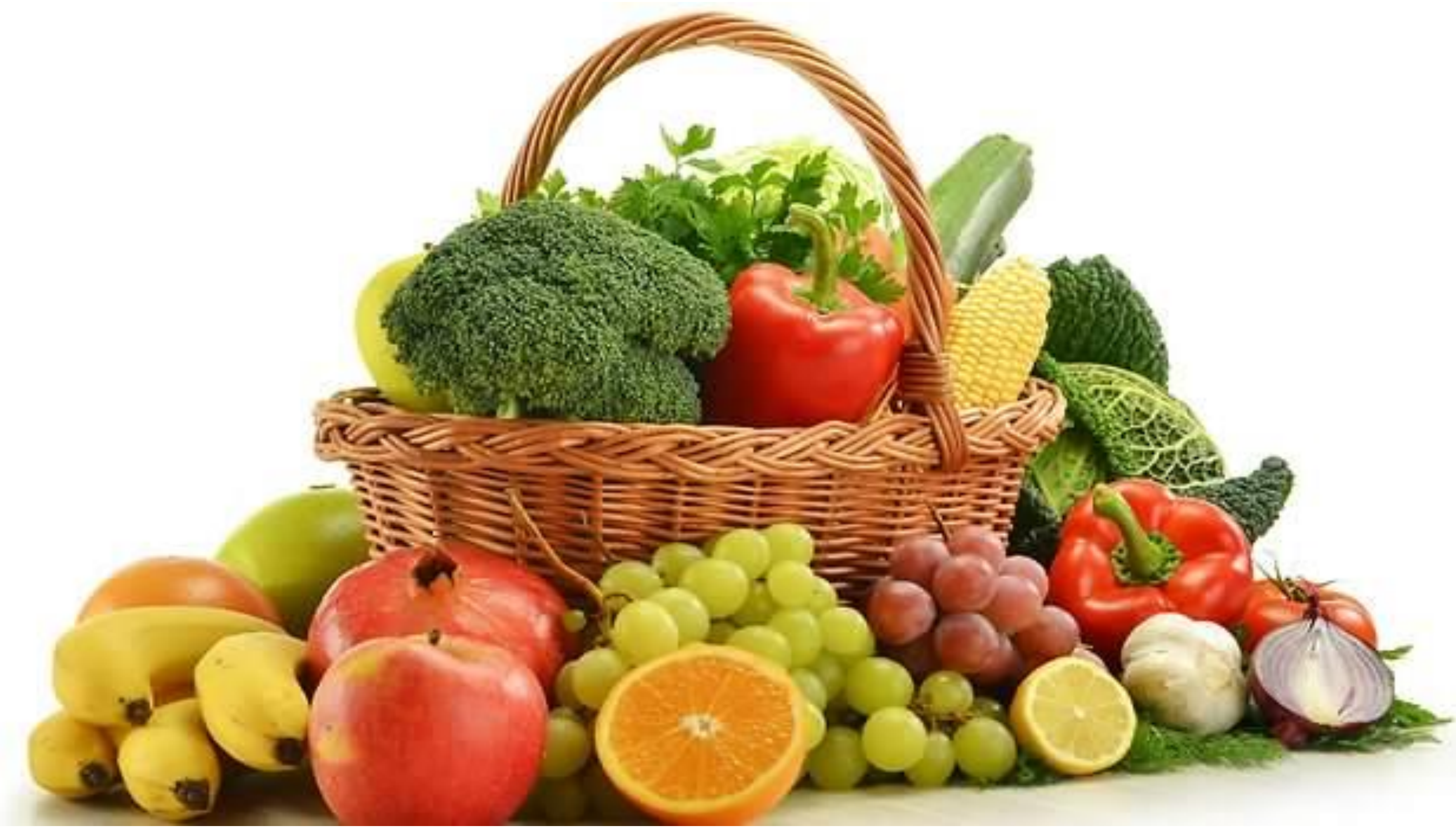

# What is a balanced diet ?

A diet that contains **adequate** amounts of all **necessary** nutrients required for healthy growth and activity

# Different food groups

**THE TEN FOOD GROUPS ARE:**

1. Grains, white roots and tubers, and plantains
2. Pulses (beans, peas and lentils)
3. Nuts and seeds
4. Dairy
5. Meat, poultry and fish
6. Eggs
7. Dark green leafy vegetables
8. Vitamin A-rich fruits and vegetables
9. Other vegetables
10. Other fruits

# SMART PLATE

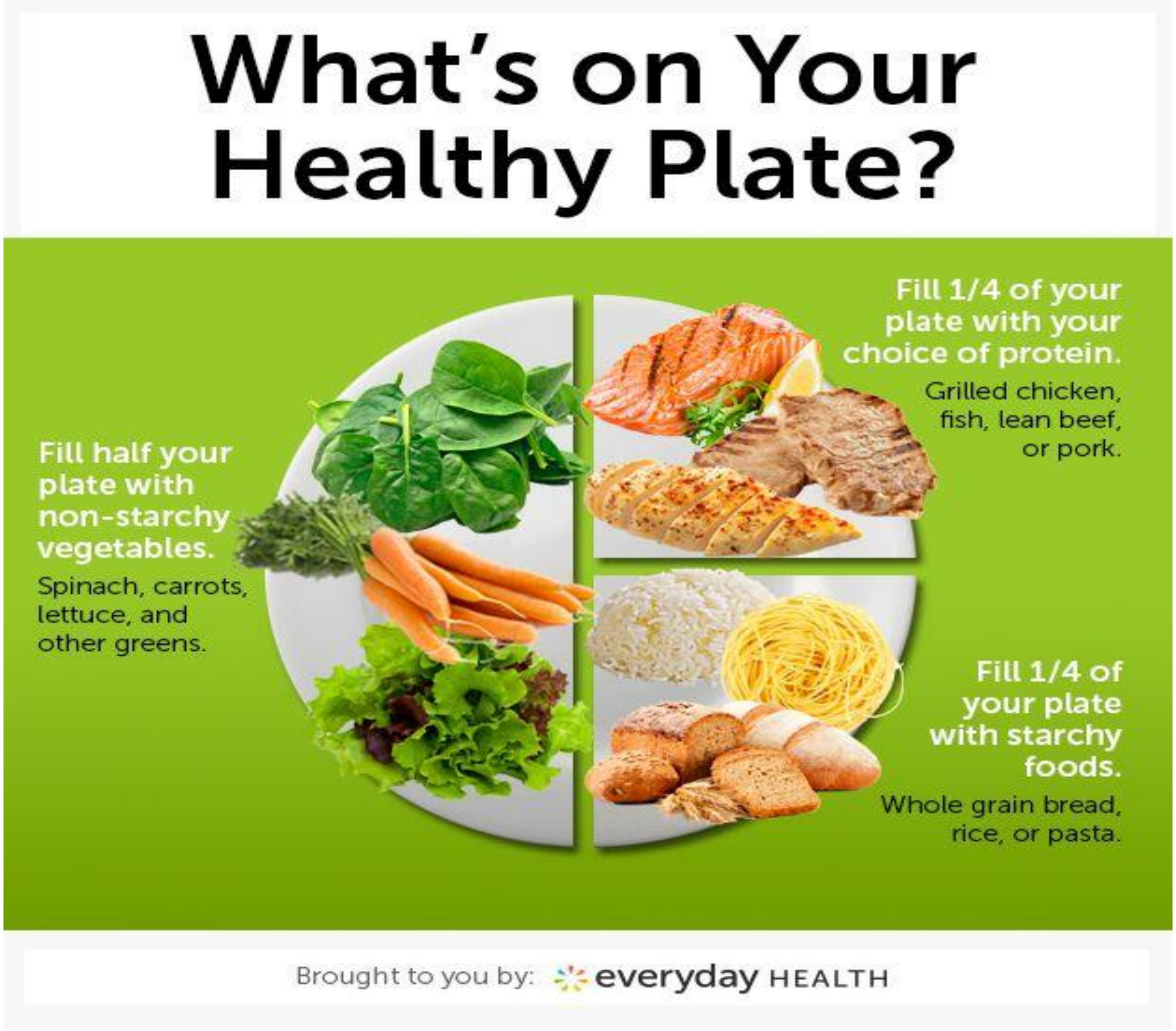
What's on Your Healthy Plate?
Fill 1/4 of your plate with your choice of protein.
Grilled chicken, fish, lean beef, or pork.
Fill half your plate with non-starchy vegetables.
Spinach, carrots, lettuce, and other greens.
Fill 1/4 of your plate with starchy foods.
Whole grain bread, rice, or pasta.
Brought to you by: everyday HEALTH

# WHAT IS JUNK FOOD ?

-Food with high calories

-Rich in salt, sugar and fat

-With little nutritive value.

-Tasty and usually cheap.

-Contains little or no fibre.

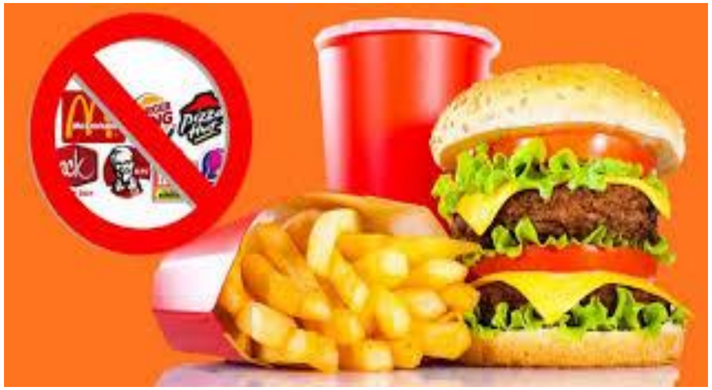

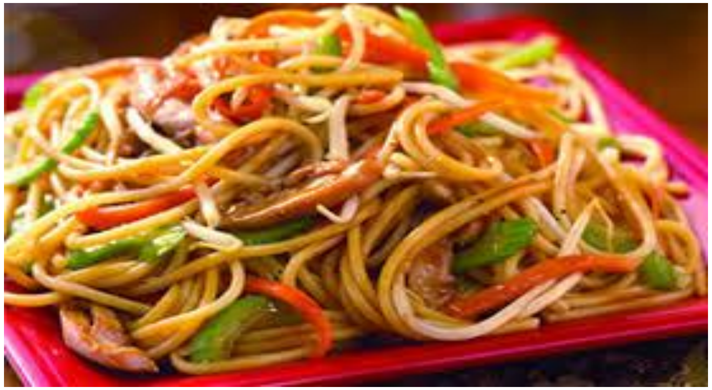

# Healthy Diet

- **Wash** vegetables & fruits properly before cooking.
- Develop the habit of taking meals at **regular intervals.**
- Use **boiling, steaming** etc. as methods of cooking, **avoid fried food**
- Increase of locally available seasonal **fruits** and **green leafy vegetables** in the diet.
- Vegetable- **3-4 serving/bowl** & **at least one fruit** daily

# Healthy Diet

- Restrict intake of ghee, oils (Recommended-**500gm/person/month**)
- **Trans-fat** (margarine, Vanaspati, Dalda) consumption should be **avoided**
- Reduce salt intake **<5gm per day**
- Avoid intake of aerated drinks (cola), fast/processed food (Chips, burger, samosa etc.).

# Physical activity/ দৈহিক পরিশ্রম

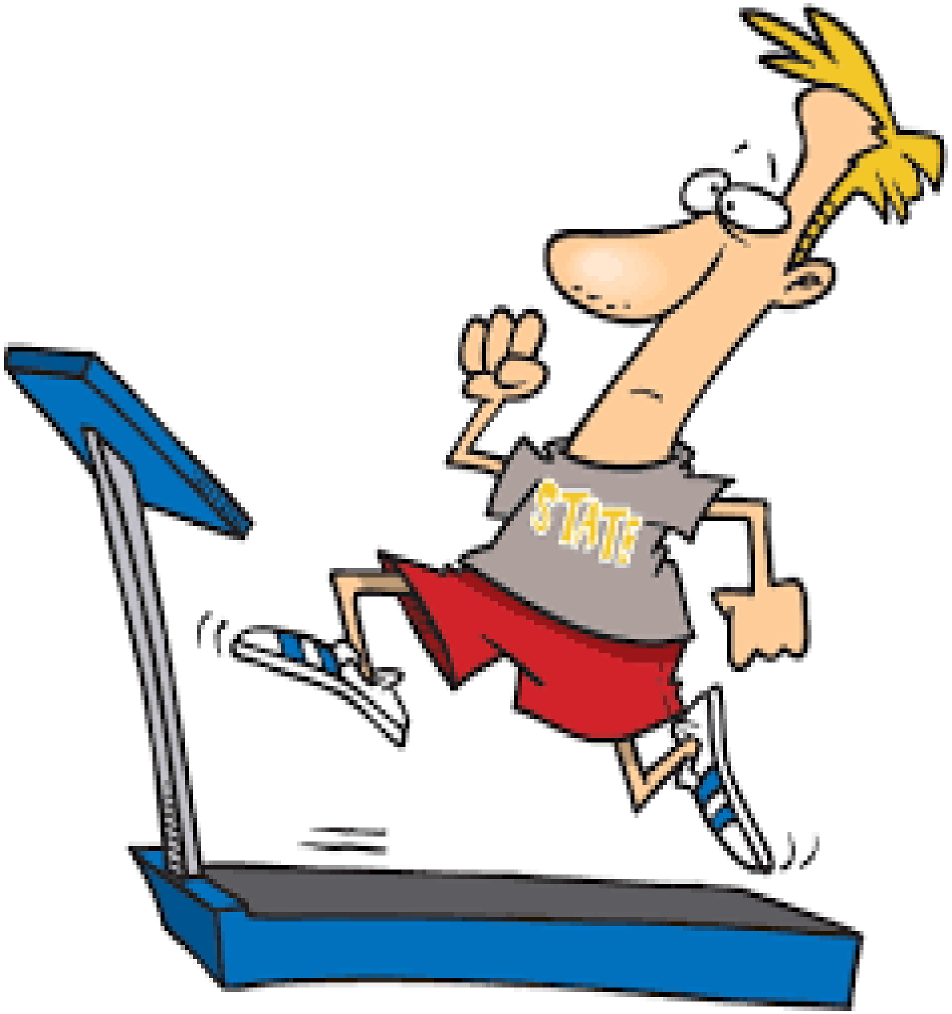


Physical activity is defined as any bodily movement produced by skeletal muscles that requires energy expenditure

# Benefits of Regular Physical Activity

- Physical Benefits

➢ Controls weight

➢ Improves immunity

➢ Improves blood circulation

➢ Strengthen muscles

➢ Strengthen heart

➢ Improves balance and posture

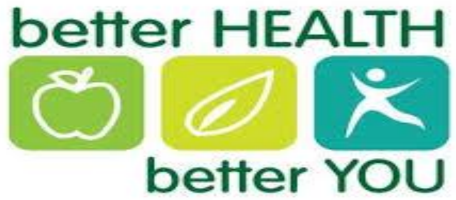


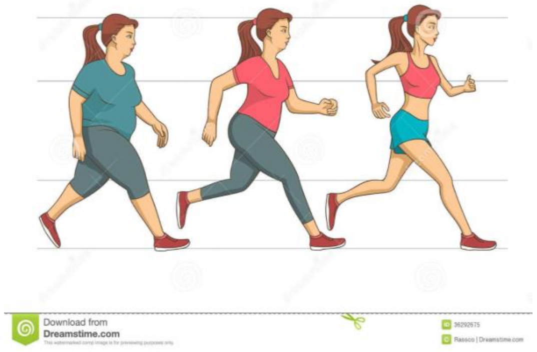

# Benefits of Regular Physical Activity

- Mental benefits:
  - Reduce stress
  - Reduce depression
  - Reduce anxiety
  - Helps to control emotions
  - Improves sleep quality
  - Improves concentration
  - Increases Social integration and bonding

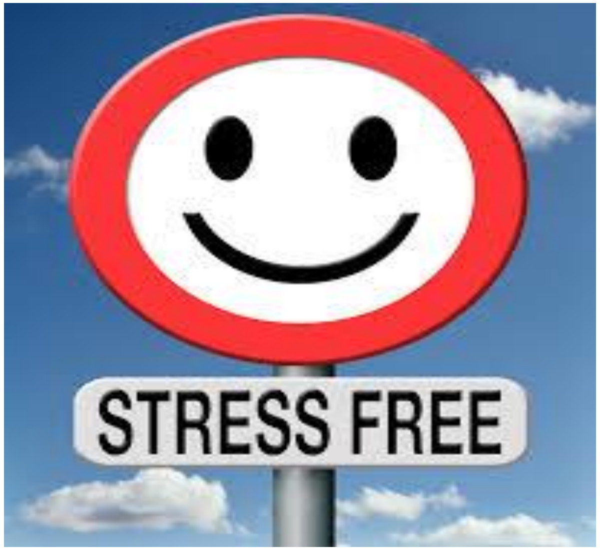

# Health Benefits of Regular Physical Activity

- Reduce risk of obesity
- Reduce your risk of cardiovascular disease
- Reduce your risk for type 2 diabetes and metabolic syndrome
- Reduce your risk of some cancers
- Strengthen your bones and muscles

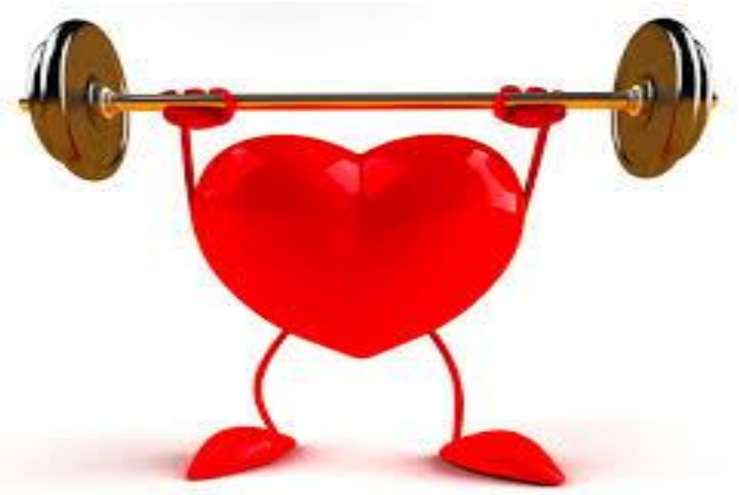

# Recommendations for Physical Activity

- **For Children 6-17 years:**

Everyday 60 minutes of moderate to vigorous PA

- **For Adults(18-64 years):**

30 minutes of moderate PA for at least 5 days/week i.e. 150 minutes a week or 25 minutes vigorous PA 3days/week i.e. 75 minutes/week

- **For Older Adults(65 years old or older):**

Similar to above- 30 minutes of moderate PA; at least 5 days/week

# What counts as moderate physical activity

Any physical activity is better than none. It is never too late to get more active to improve health. Activities could include:

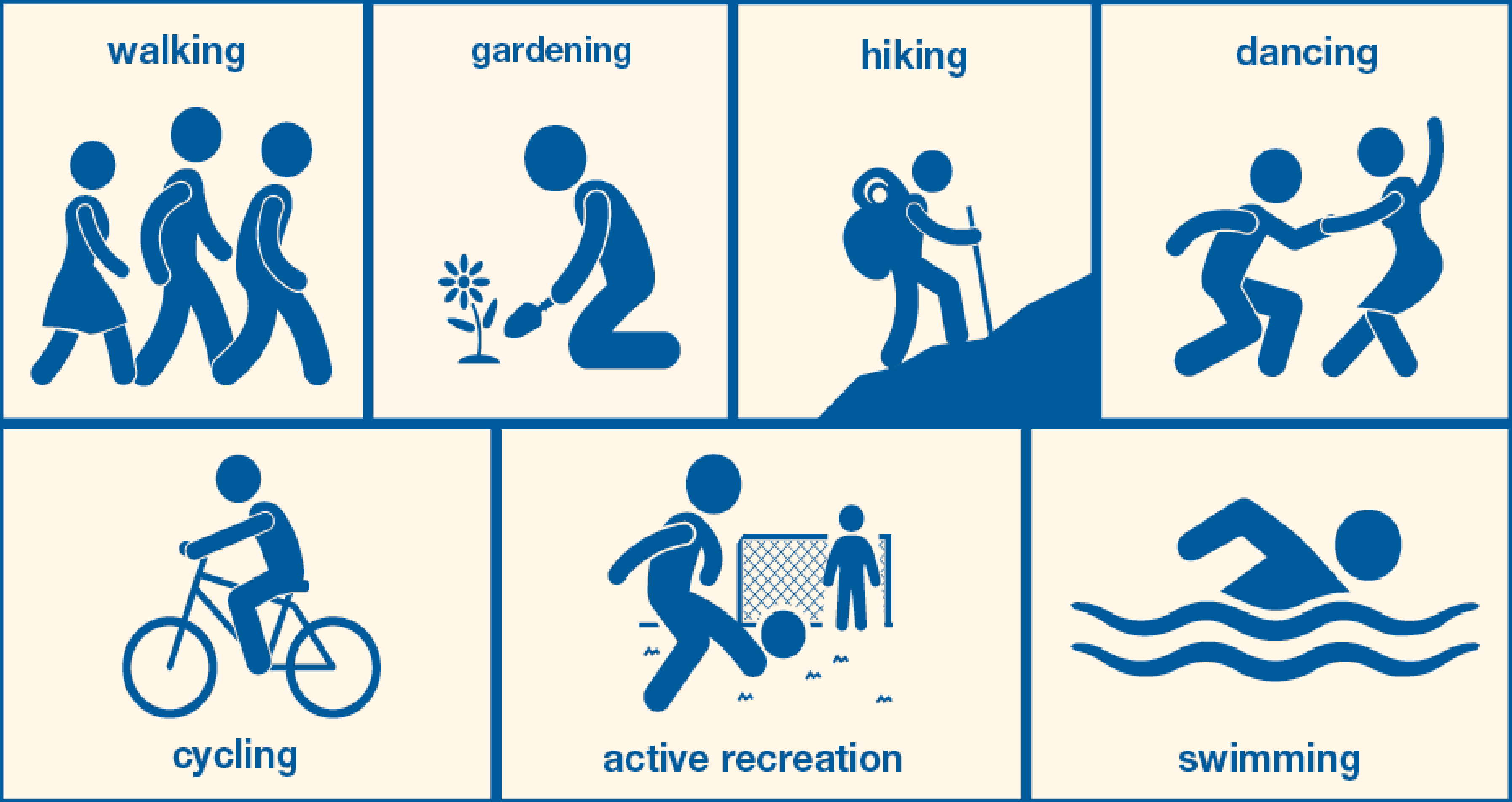

# Myths vs Facts

- Myth: Consumption of junk food and fast food leads to indigestion but has no relationship with CVD

- Fact: Junk food and Fast food increases the risk of CVD

# Myths vs Facts

- Myth: Only exercising in gym will lower a person's chance of developing CVD
- Fact: Brisk walking, cycling, swimming, gardening, aerobic (free hand exercise) are equally good

# Myths vs Facts

- Myth: Only exercising in gym will lower a person’s chance of developing CVD
- Fact: Brisk walking, cycling, swimming, gardening, aerobic (free hand exercise) are equally good

# “I am a housewife. I don’t get time to exercise.” or, “I do lot of work in house, I don’t need any extra exercise”

- It can be done in the evening also.
- Only 30 minutes is required from 24 hours.
- That too can be done in 3 bouts of 10 minutes also.

# “I am old, I have knee and back pain”

- It can be done in the evening also.
- Only 30 minutes is required from 24 hours.
- That too can be done in 3 bouts of 10 minutes also.

# Sedentary habits

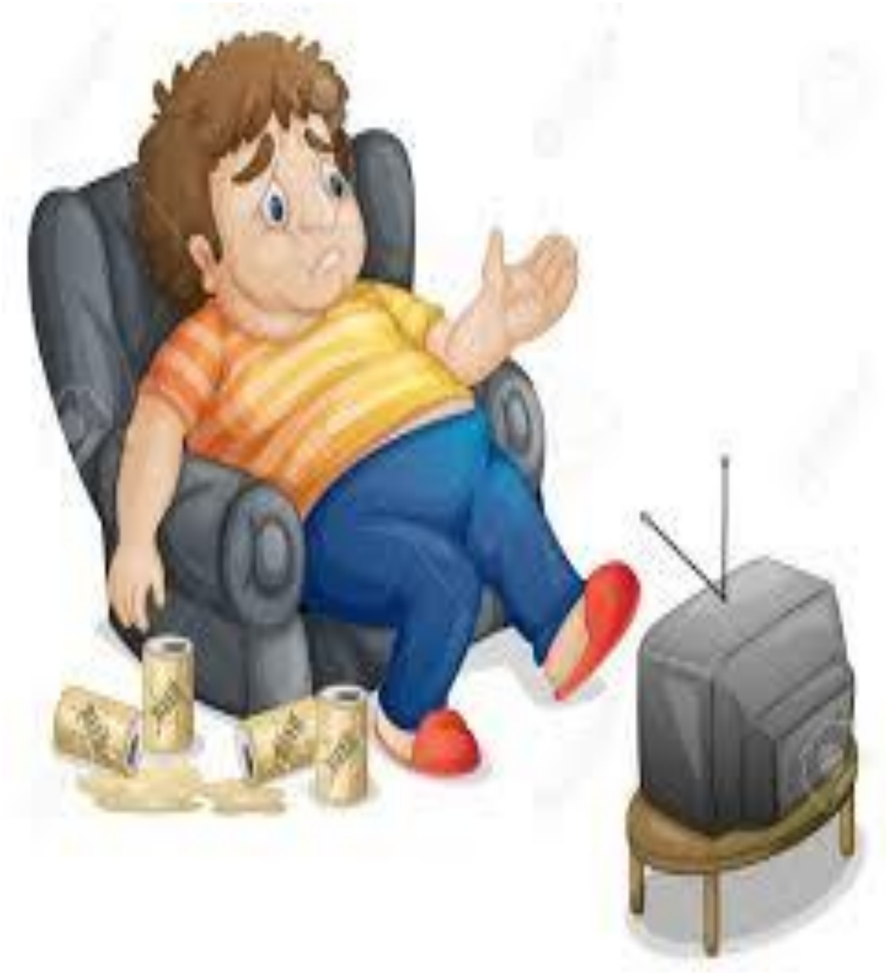

- Watching TV for long duration
- Computer work/ surfing internet
- Use of smartphones/ mobile
- Sitting and gossiping
- Excessive sleeping
- Job related- Teachers, IT professionals, doctors, lawyers etc.

# Attention!!!

- **Screen time use** (TV, Computer, Mobile etc.) should be restricted to **2 hours/day**
- **Sleep** at least **7-8 hours/day**
- **Sleep deprivation** is also a risk factor for obesity, CVDs and metabolic syndrome

# ADDICTION

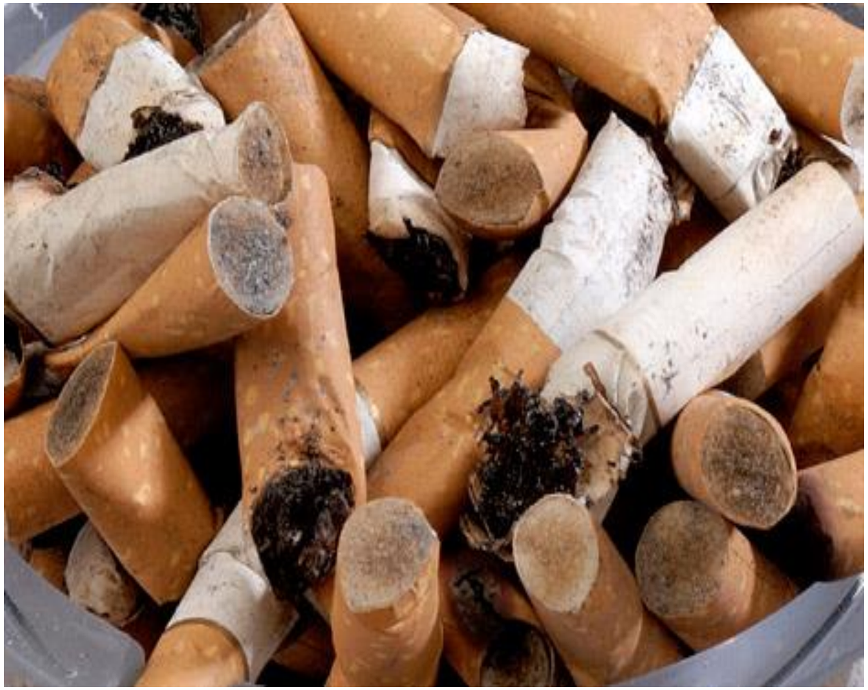

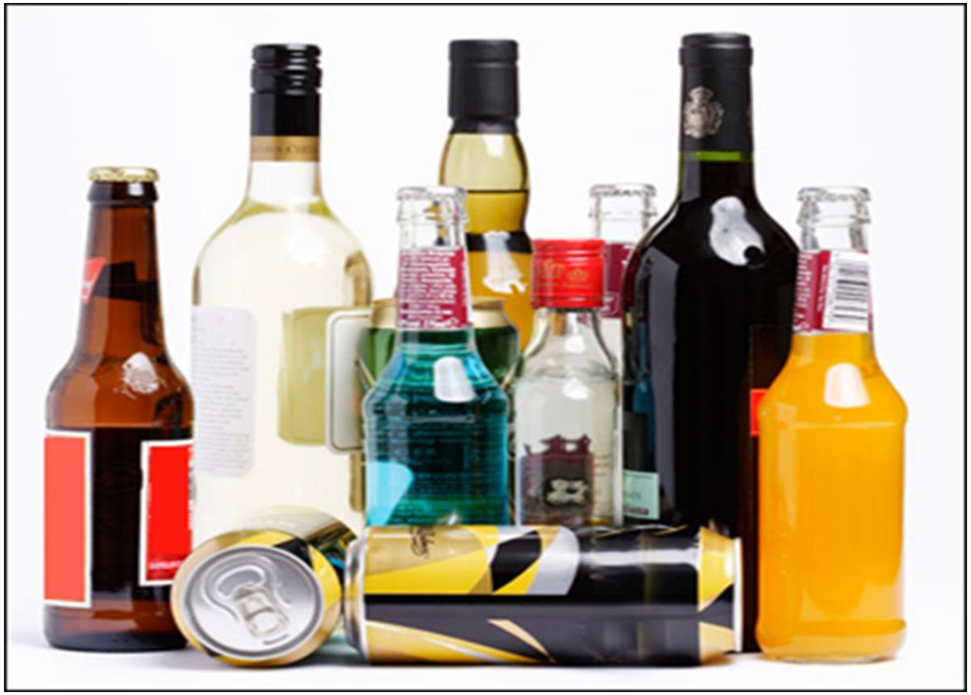

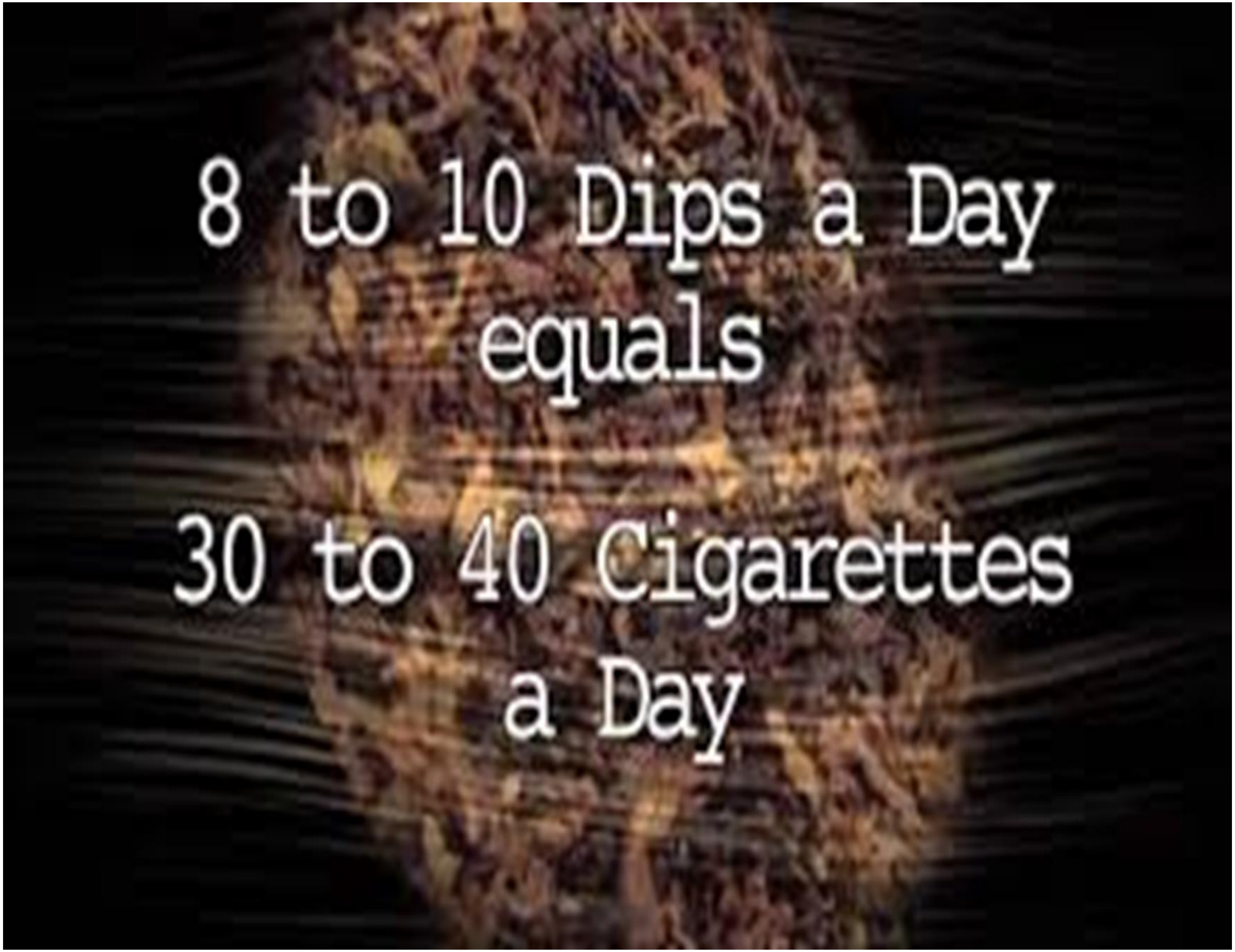
8 to 10 Dips a Day
equals
30 to 40 Cigarettes
a Day

# 2nd Hand smoking

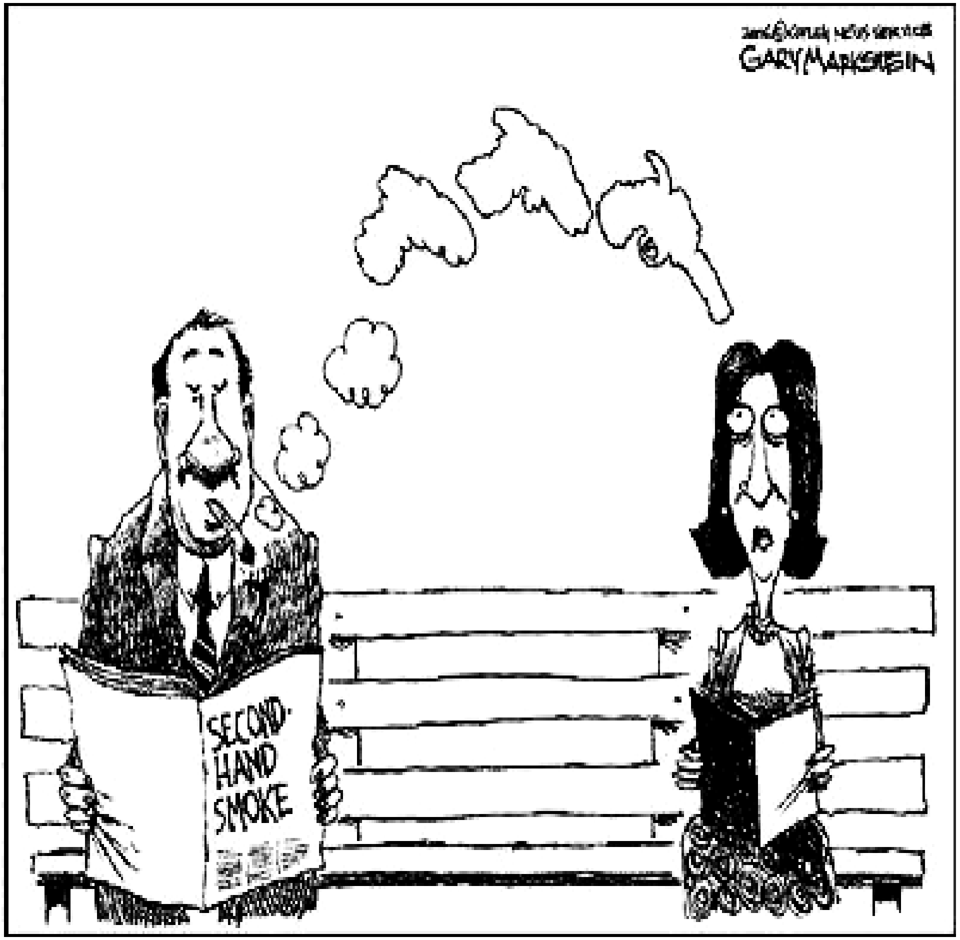


http://www.cagle.com/politicalcartoons/PCcartoons/markstein.asp

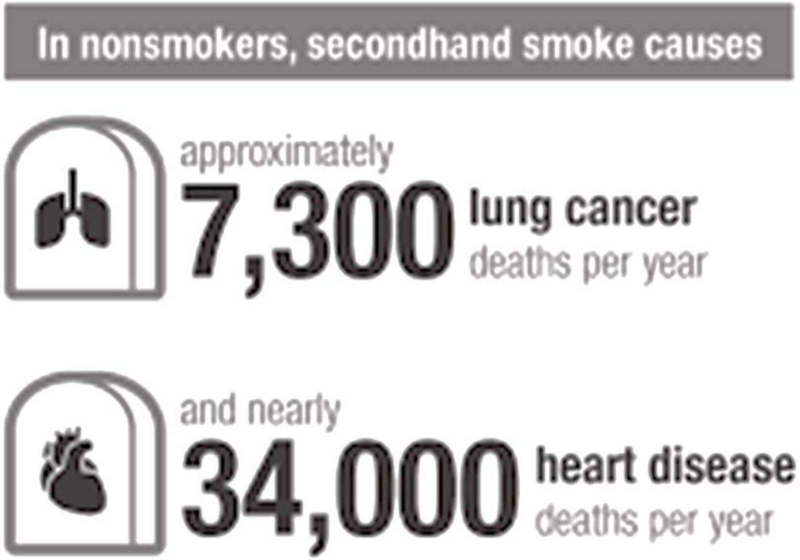

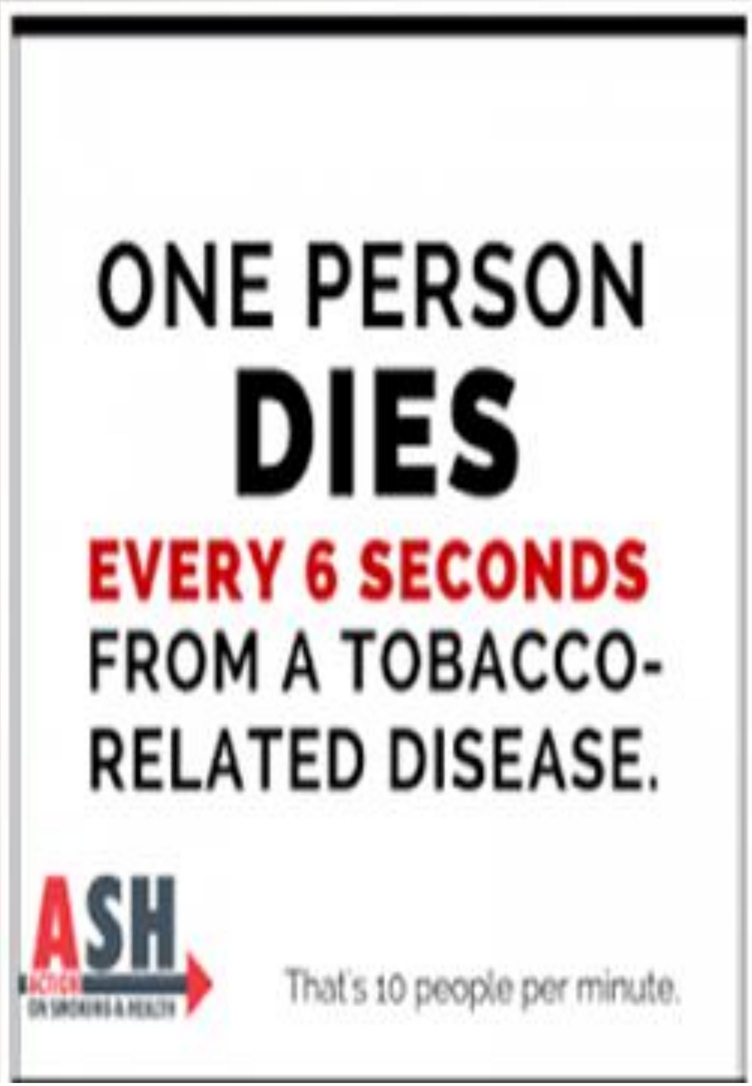
ONE PERSON
DIES
EVERY 6 SECONDS
FROM A TOBACCO-
RELATED DISEASE.
ASH
That's 10 people per minute.

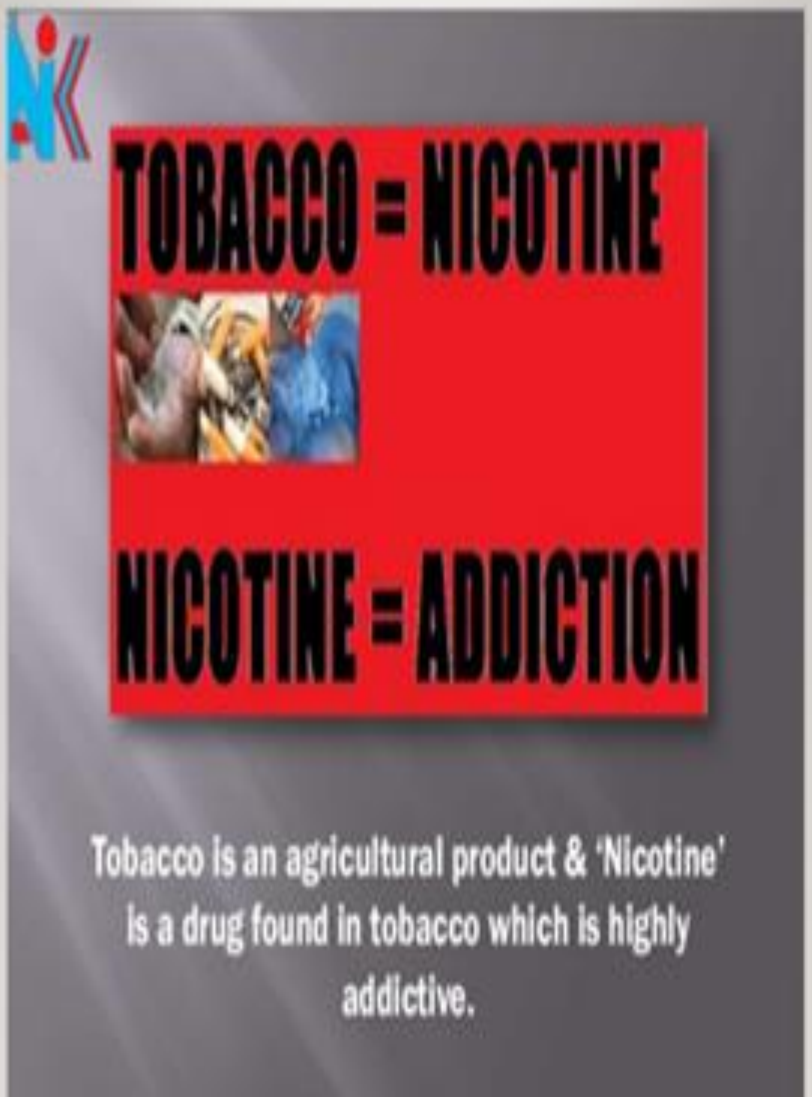
TOBACCO = NICOTINE
NICOTINE = ADDICTION
Tobacco is an agricultural product & 'Nicotine' is a drug found in tobacco which is highly addictive.

# Effects of nicotine

- Addictive
- Stimulates nervous system
- Constricts blood vessels
- Increase heart rate and blood pressure

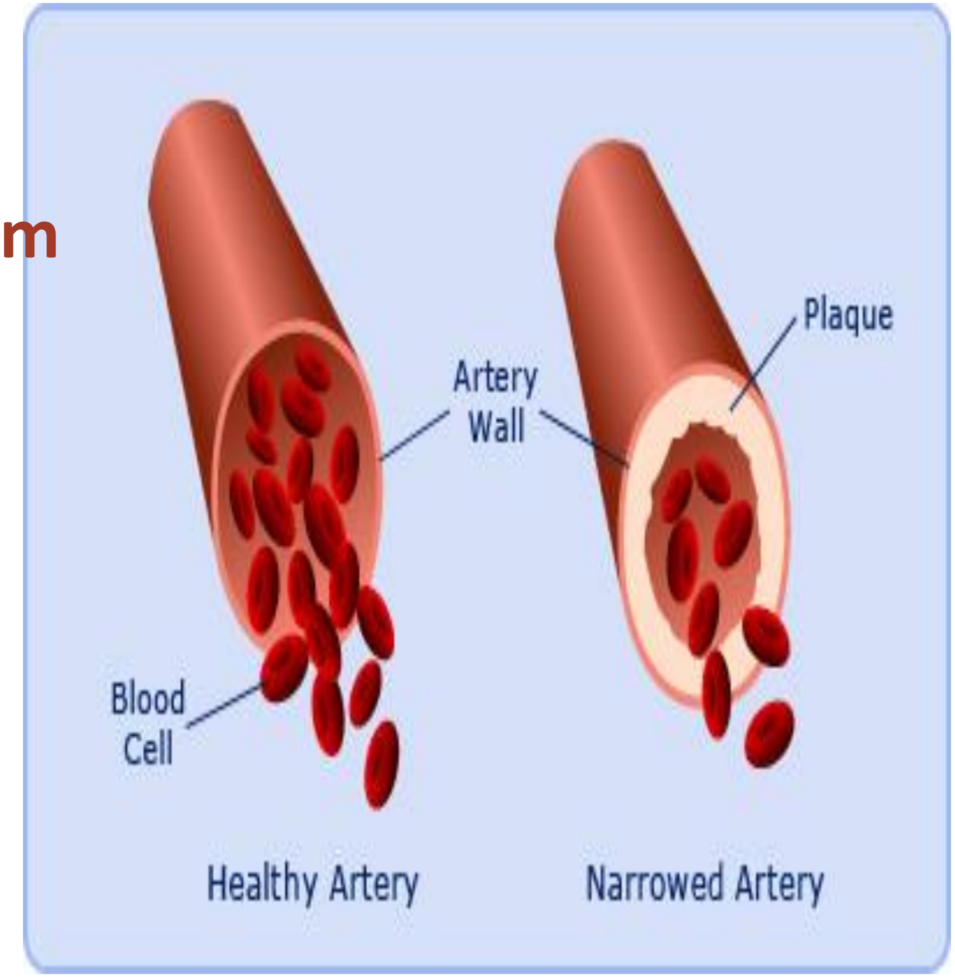

# HEART ATTACK

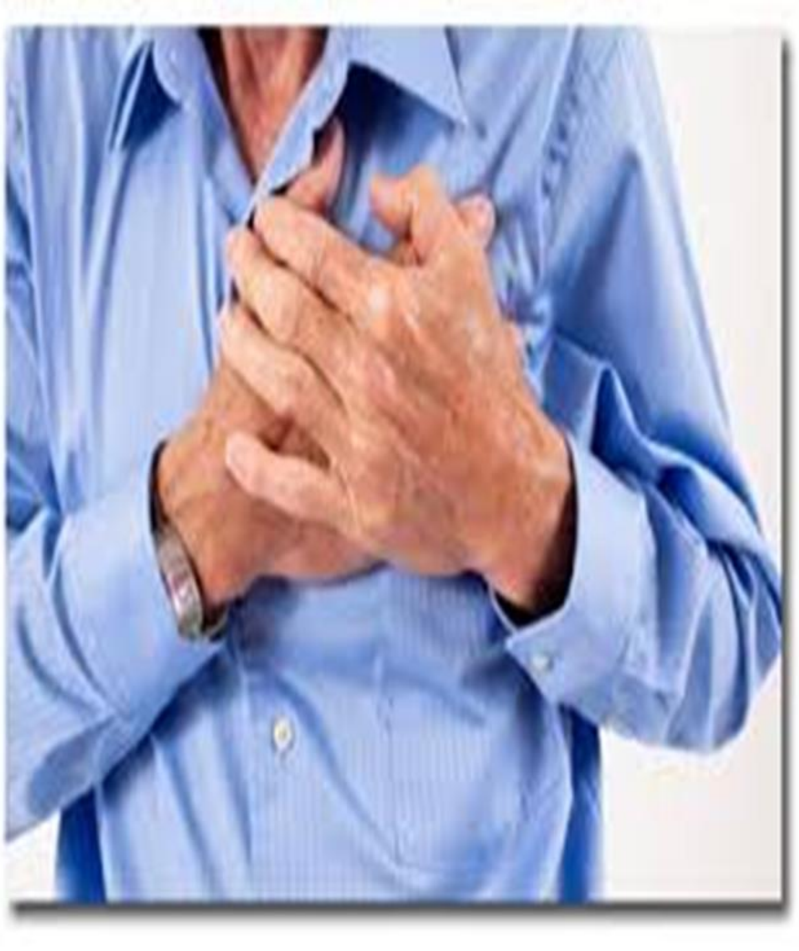

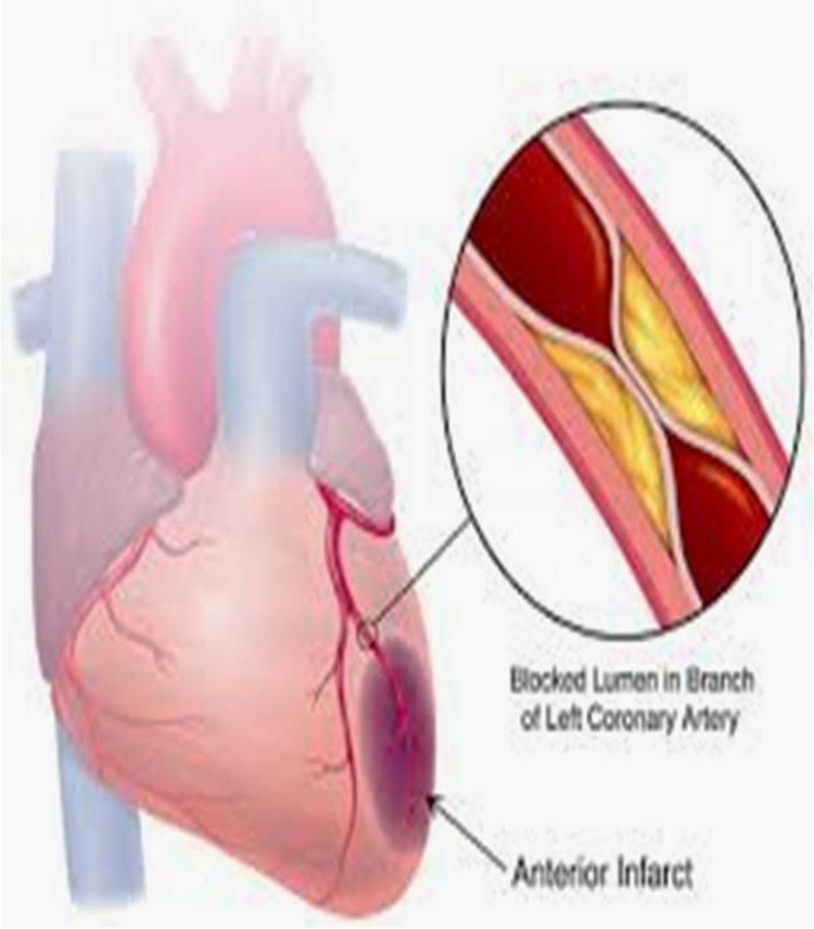

# Stroke

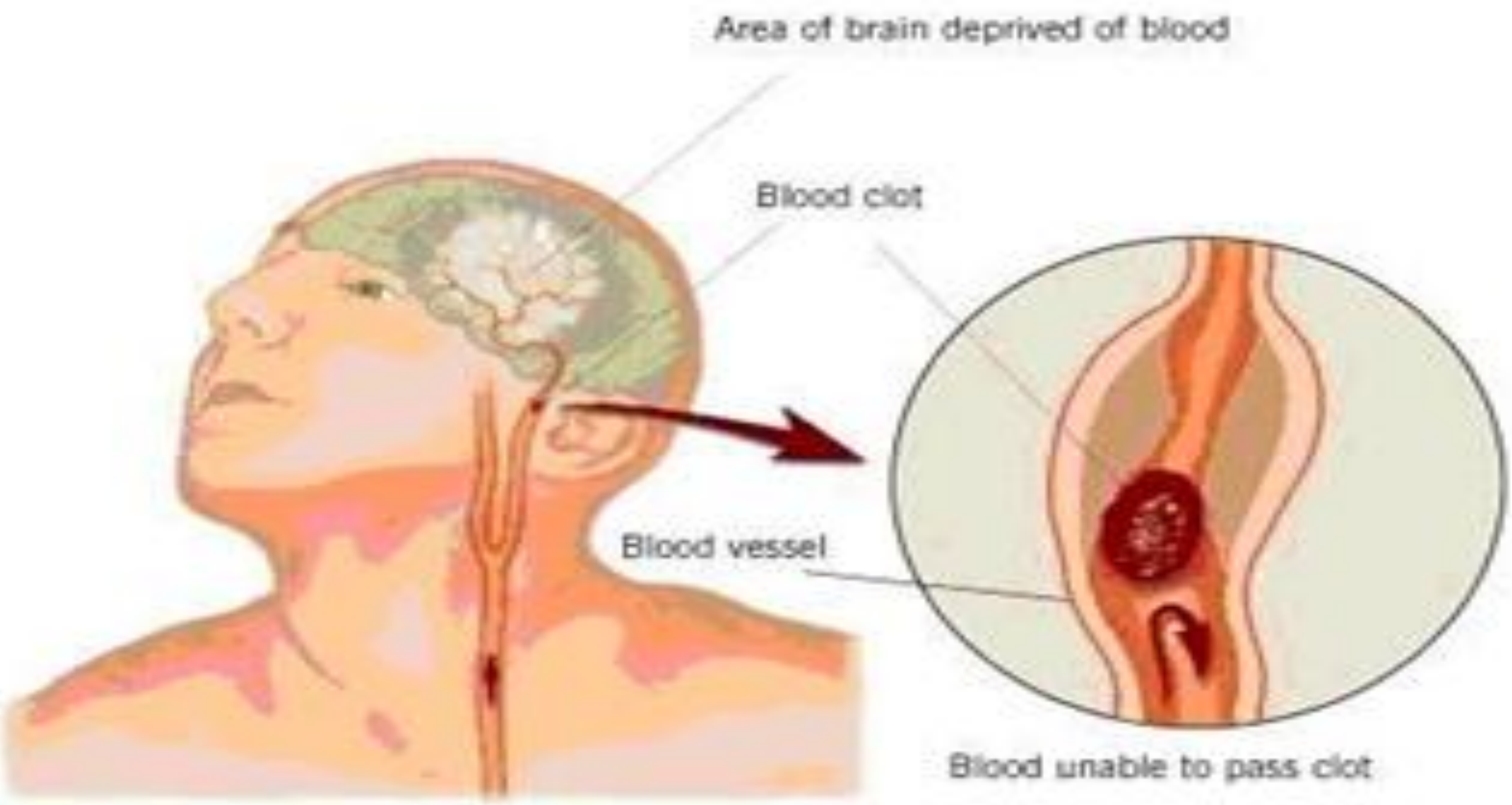

Area of brain deprived of blood
Blood clot
Blood vessel
Blood unable to pass clot

# Tar deposition in lung

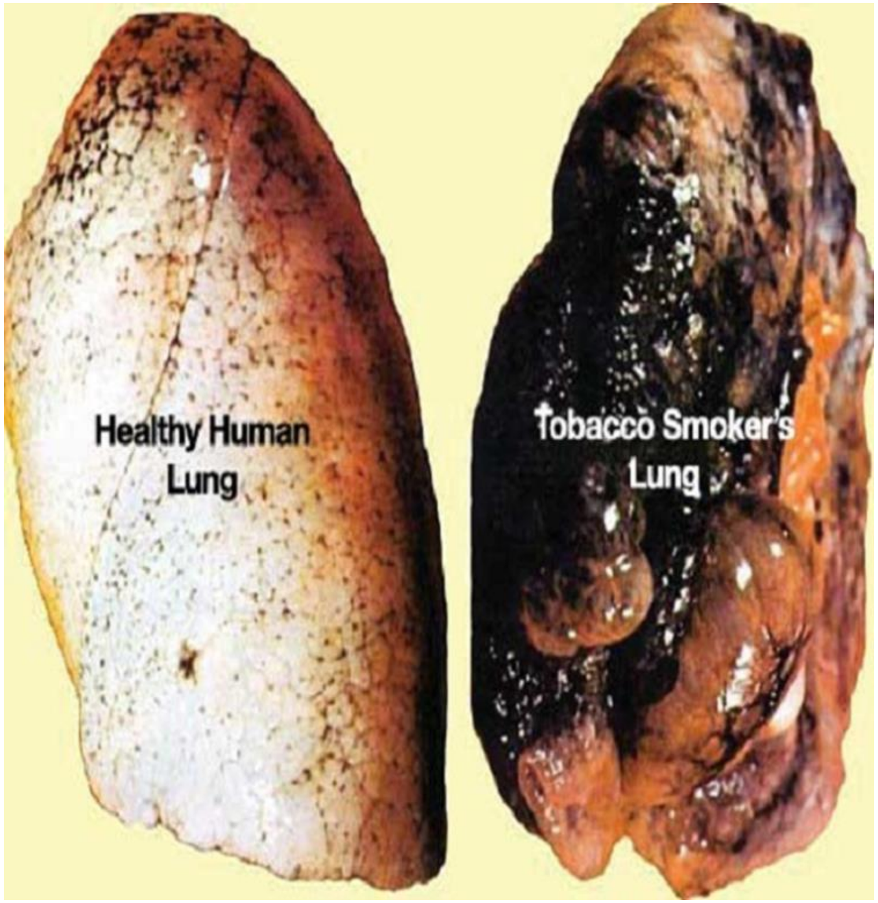


- Smokers are 10 times more likely to get lung cancer and emphysema compared to non smokers.

Oral cancer

Respiratory problems

## Other Hazards of Tobacco

Dental caries

Cataract

# HOW TO Quit

This is the one and only
way for quitting

# Alcohol abuse

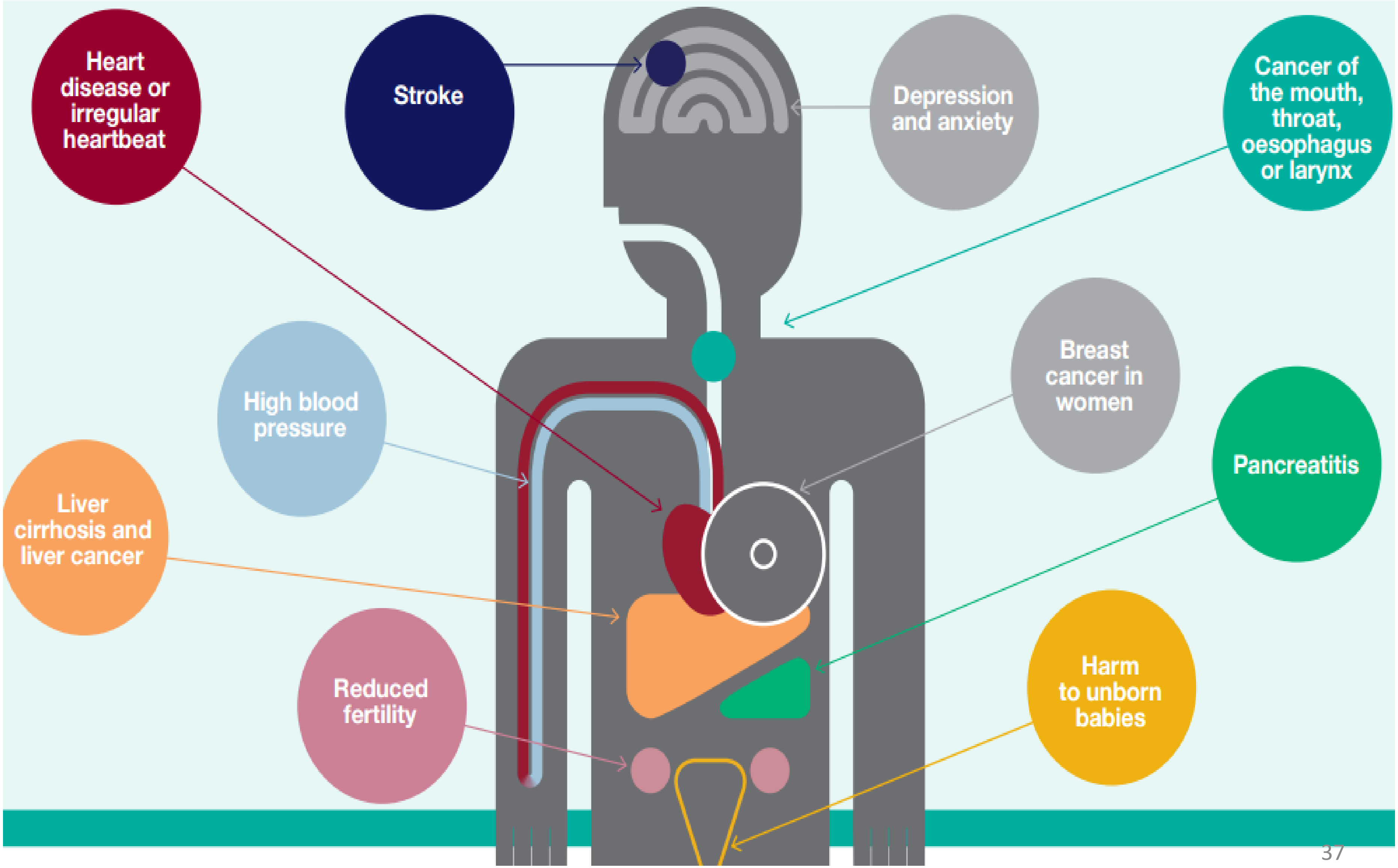

# What is moderate drinking?

- **3-4 standard drinks** per drinking episode, no more than 9 drinks per week for women and 12-14 for men. Also, moderate drinking means limiting **how fast you drink**

- A standard drink is equal to-

✓ 355 ml. beer with 5% alcohol

✓ 150 ml. glass of wine (12.5% alcohol).

✓ 45 ml. of 80 proof liquor (40% alcohol)

# If you're thinking about moderate drinking for yourself!

- If you've had significant alcohol-related problems and **are currently not drinking**, trying moderate drinking may put you at risk again for alcohol-related problems.

- If you're currently drinking more than the guidelines of moderation, you will reduce your risk for problems by cutting back.

# HYPERTENSION

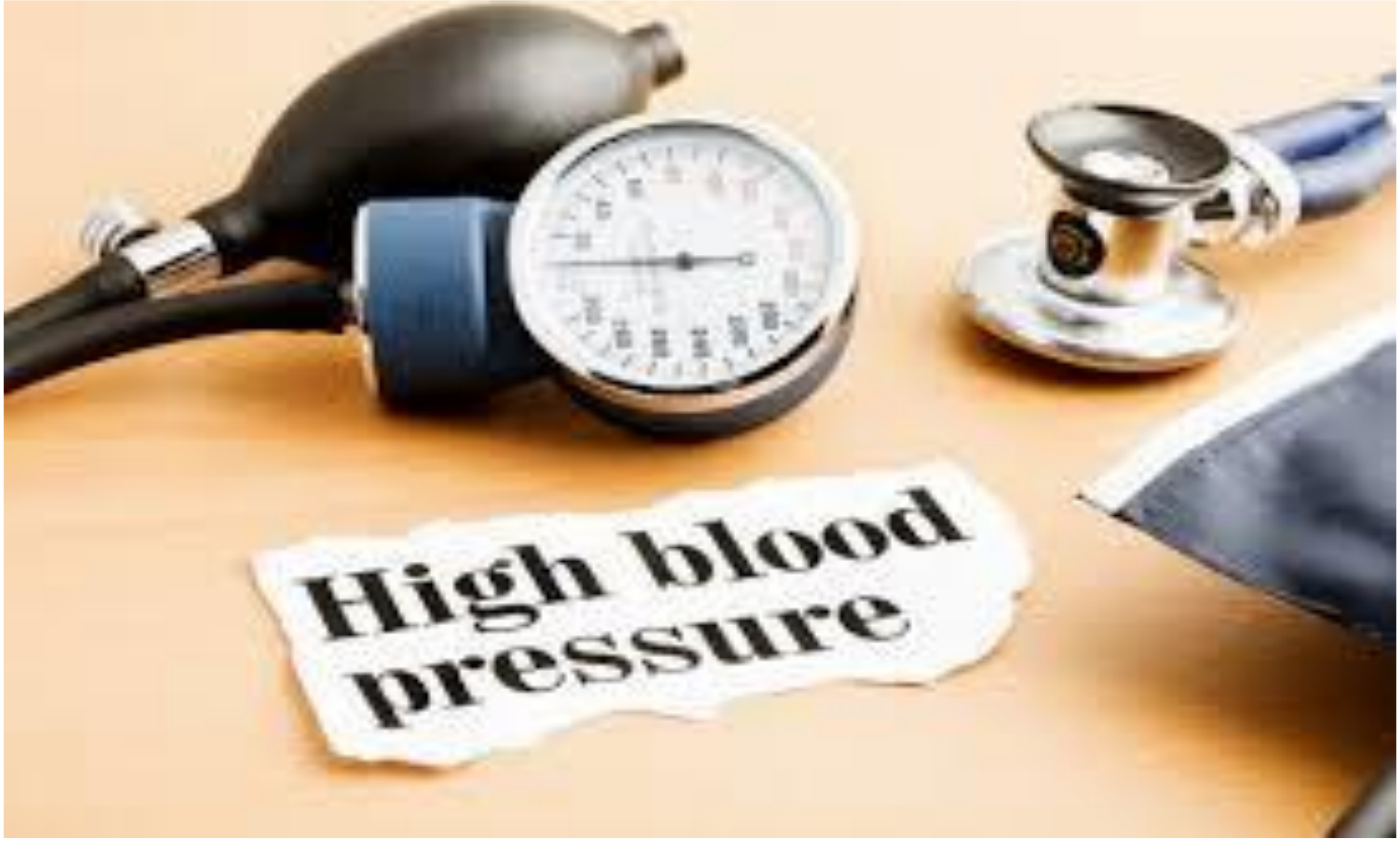

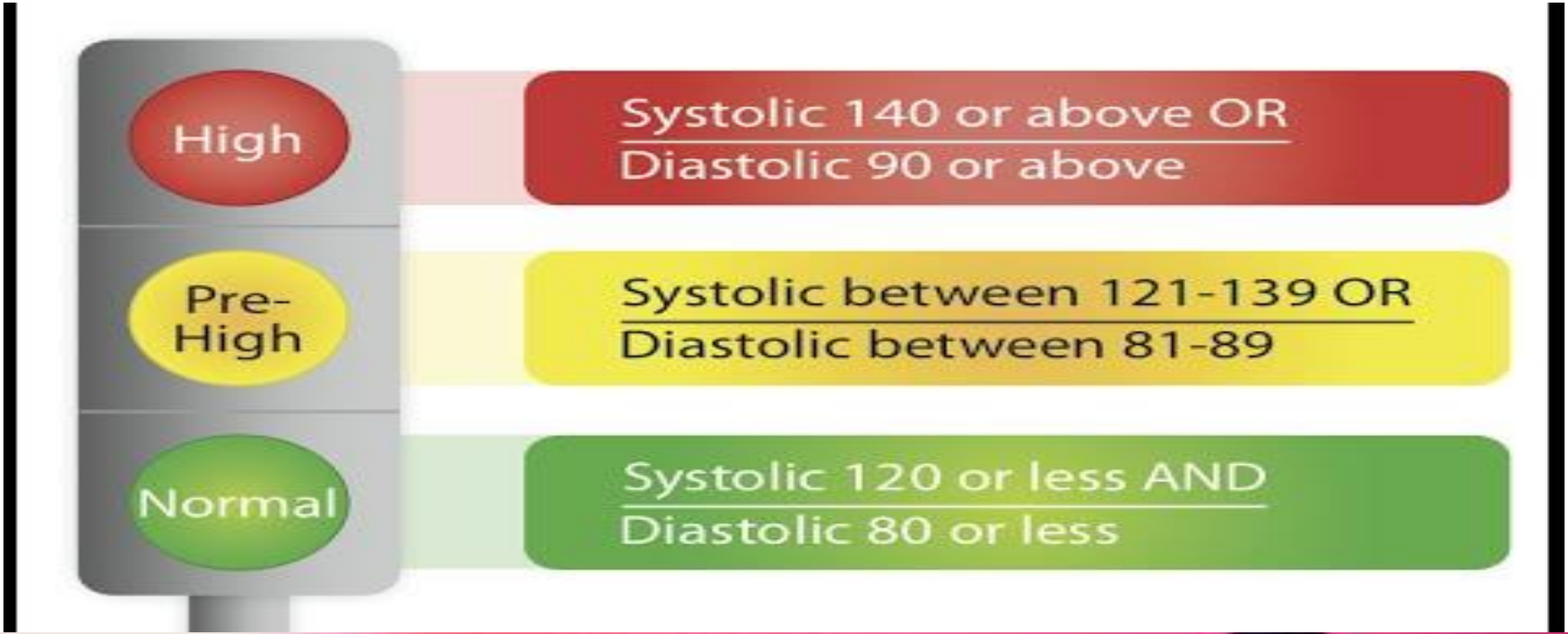
High
Systolic 140 or above OR
Diastolic 90 or above
Pre-High
Systolic between 121-139 OR
Diastolic between 81-89
Normal
Systolic 120 or less AND
Diastolic 80 or less

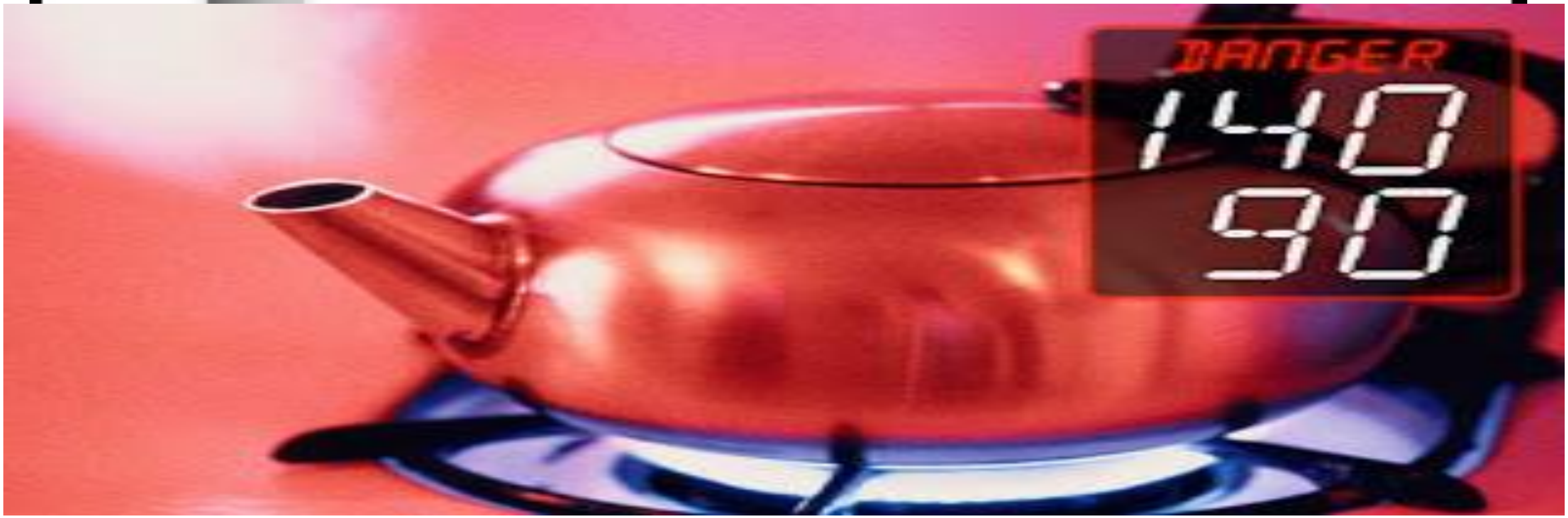
DANGER
140
90

# RISK FACTORS

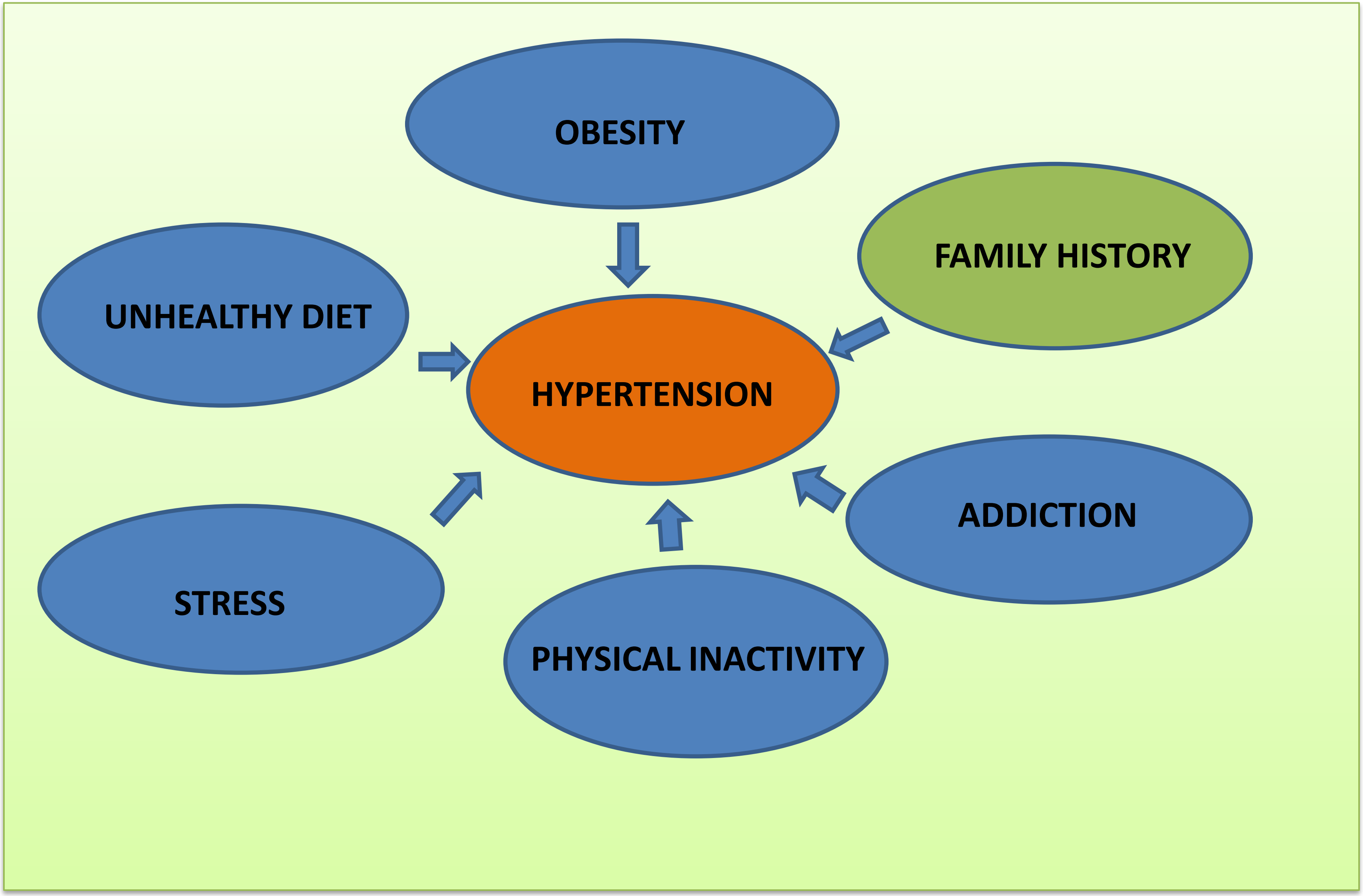

# Hypertension self care

- Self monitoring
- Compliance to antihypertensive drug

**"If I start taking drugs I have to take life long!"**

- It is better to start drugs to control BP rather than let hypertension damage your other organs like heart and kidney permanently.
- There is always chance of stopping the drug **(on doctor's advice)** if your BP is lowered.

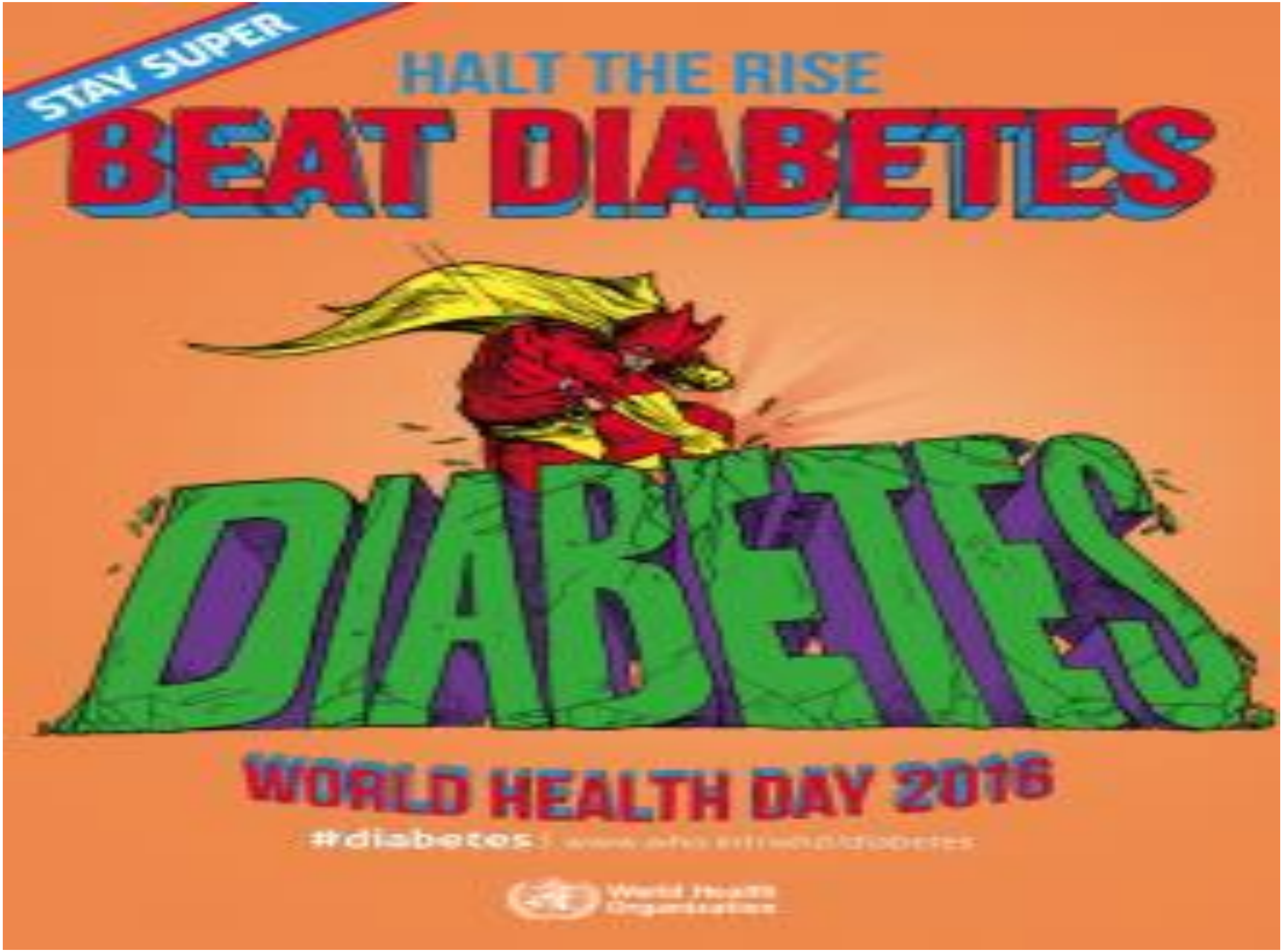
STAY SUPER
HALT THE RISE
BEAT DIABETES
DIABETES
WORLD HEALTH DAY 2016
#diabetes
World Health Organization

# Diabetes is a huge and growing problem…

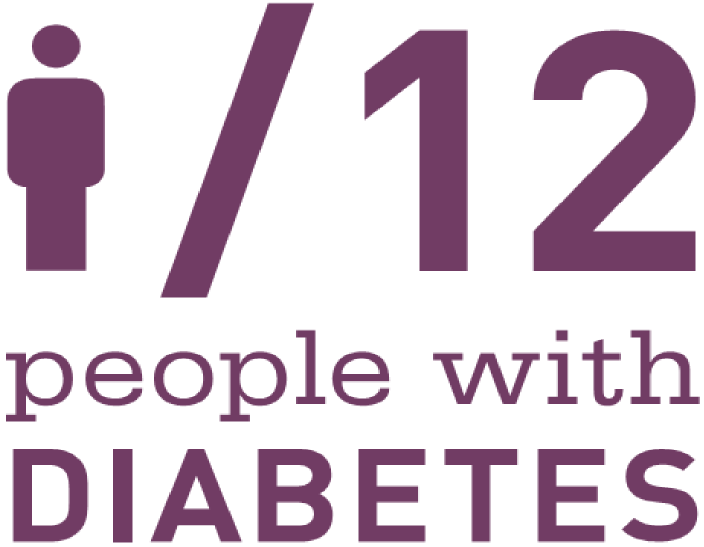


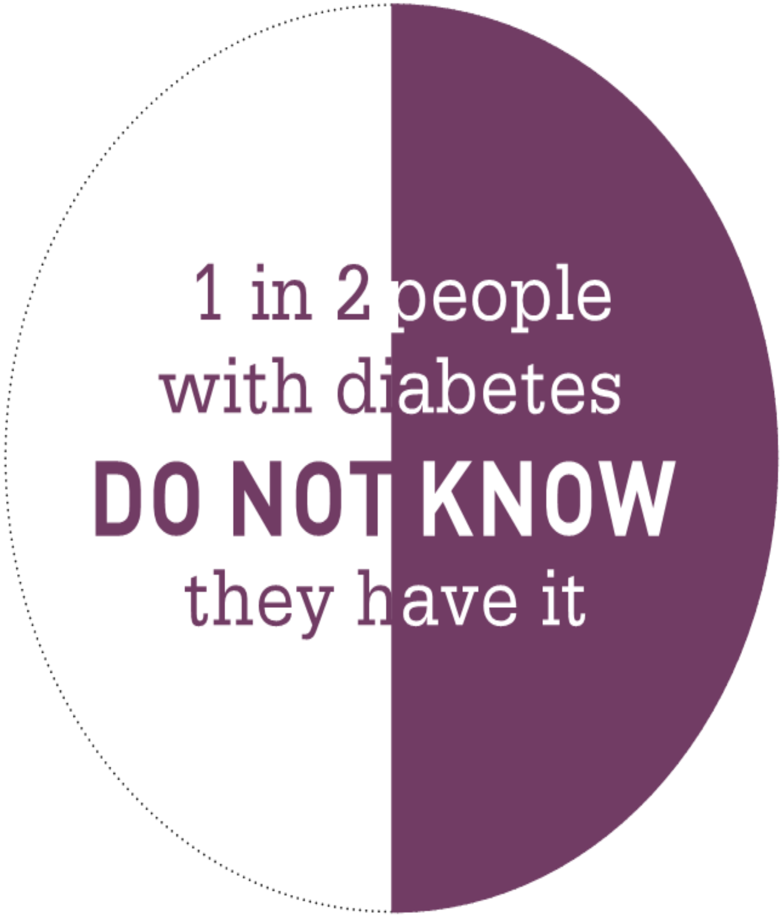

# DIABETES
## RISK FACTORS

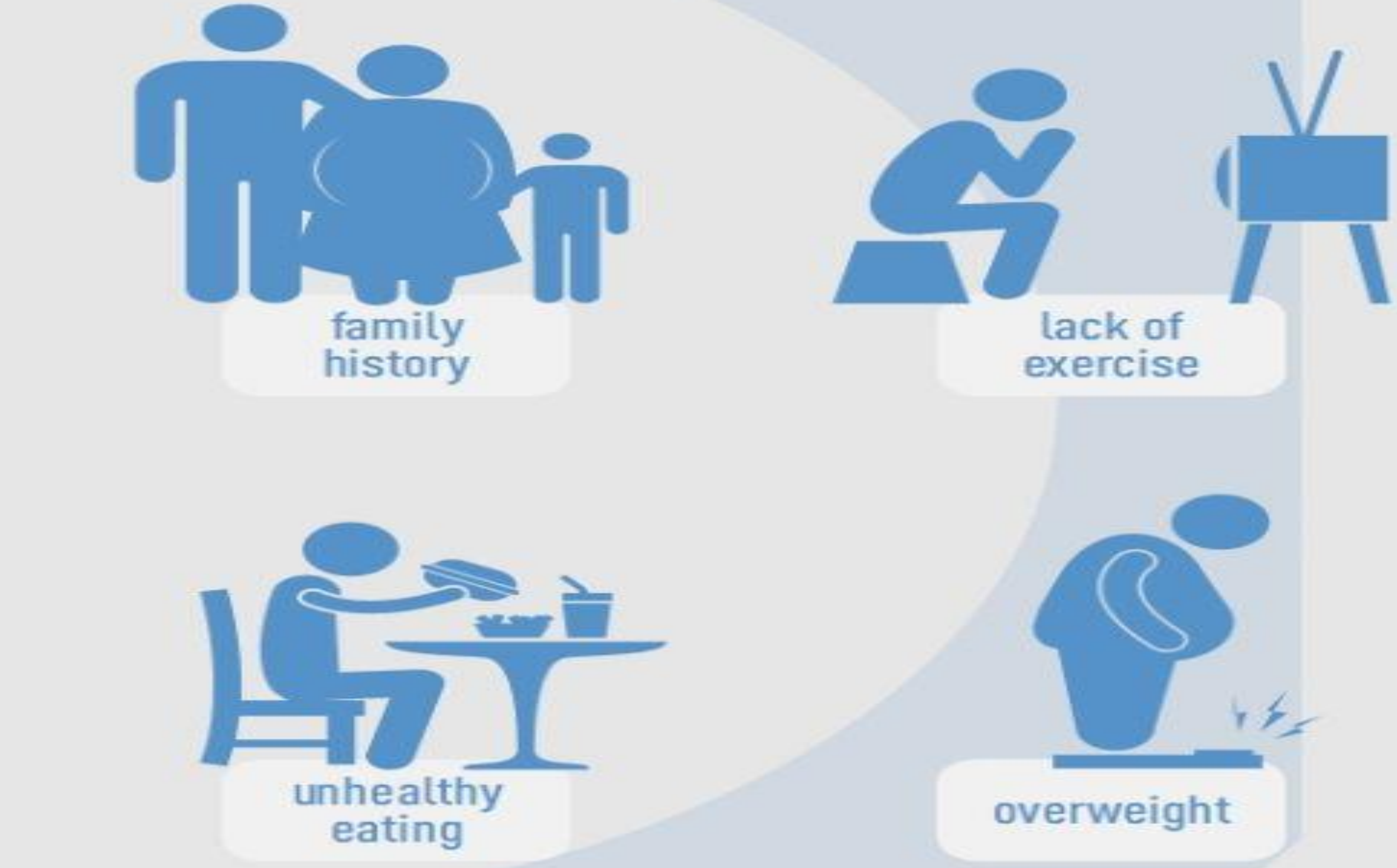

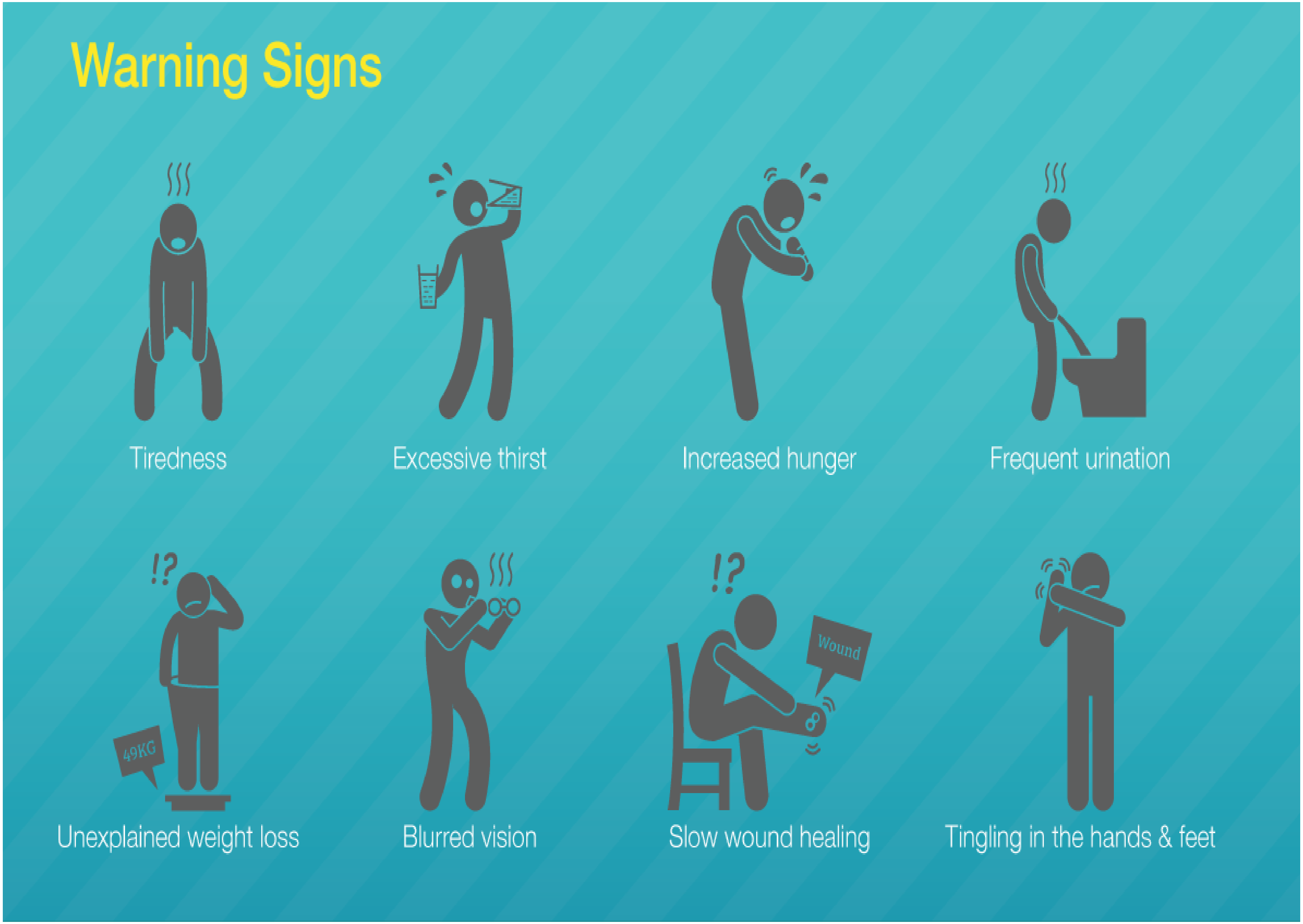
Warning Signs
Tiredness
Excessive thirst
Increased hunger
Frequent urination
49KG
Unexplained weight loss
Blurred vision
Wound
Slow wound healing
Tingling in the hands & feet

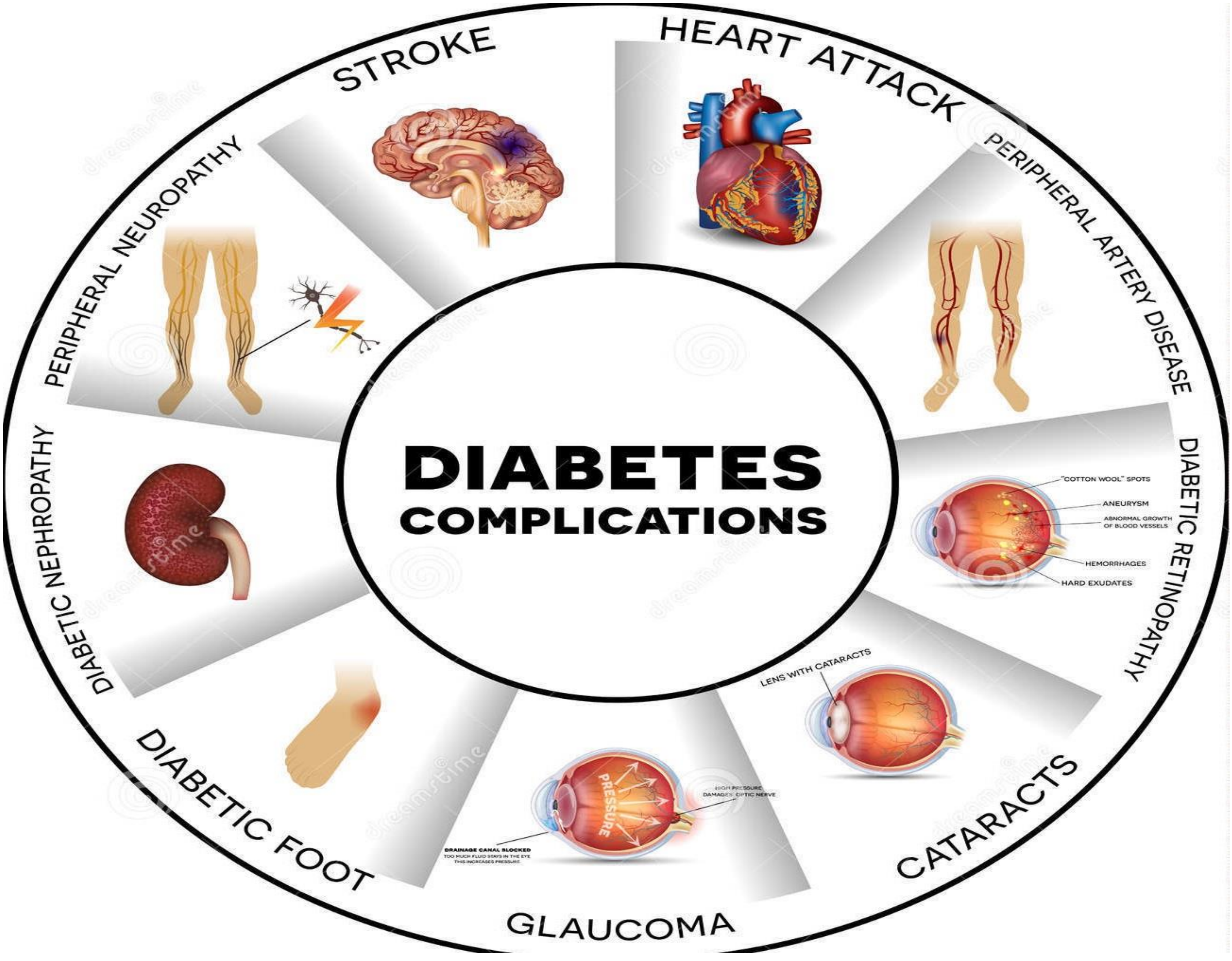

DIABETES
COMPLICATIONS
STROKE
HEART ATTACK
PERIPHERAL ARTERY DISEASE
DIABETIC RETINOPATHY
"COTTON WOOL" SPOTS
ANEURYSM
ABNORMAL GROWTH OF BLOOD VESSELS
HEMORRHAGES
HARD EXUDATES
CATARACTS
LENS WITH CATARACTS
GLAUCOMA
PRESSURE
HIGH PRESSURE DAMAGES OPTIC NERVE
DRAINAGE CANAL BLOCKED
TOO MUCH FLUID STAYS IN THE EYE
THIS INCREASES PRESSURE
DIABETIC FOOT
DIABETIC NEPHROPATHY
PERIPHERAL NEUROPATHY

# American Diabetes Association, 2011
# Diagnostic criteria for Diabetes Mellitus

| | Normal Glucose tolerance | Impaired Glucose Tolerance 'PREDIABETES' | **Diabetes Mellitus** |
|---|---|---|---|
| **Fasting plasma glucose** | <100mg/dl | 100-125mg/dl | ***>/=126mg/dl*** |
| **2 hr plasma glucose during an OGTT**** | <140mg/dl | 140-199mg/dl | ***>/=200mg/dl*** |
| **Random Blood glucose + Symptoms of diabetes*** | | | ***>/= 200mg/dl*** |
| **A1C** | <5.6% | 5.7-6.4% | ***>/= 6.5%*** |

*polyuria, polydispsia, weight loss

**after a glucose load of 75g anhydrous glucose dissolved in water

YOU can control diabetes with a healthy liefstyle, proper nutrition and support.

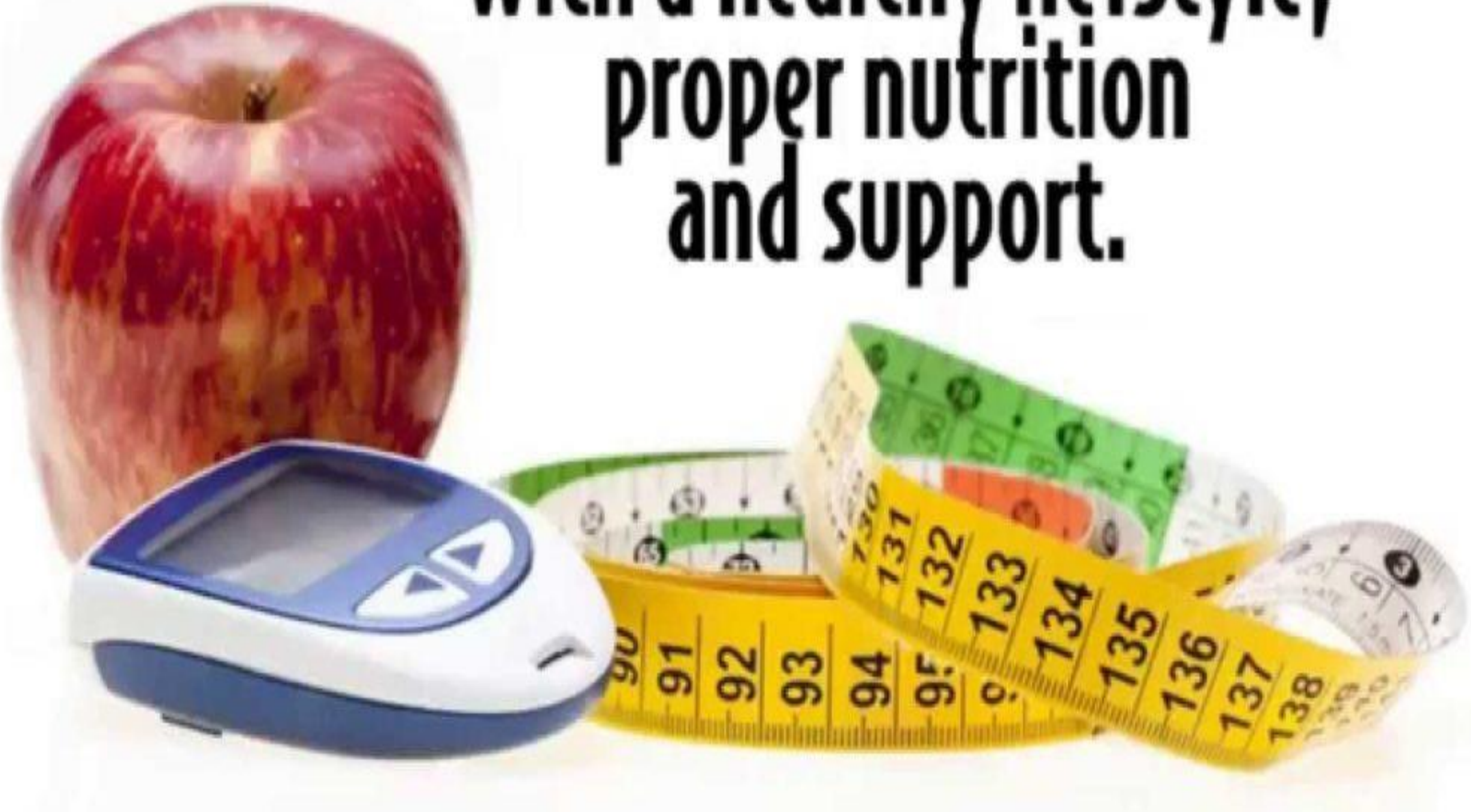

# Diabetes Self-care

- Diet
- Physical activity
- Blood sugar monitoring
- Taking drugs regularly
- Foot care
- Eye care

# Self care

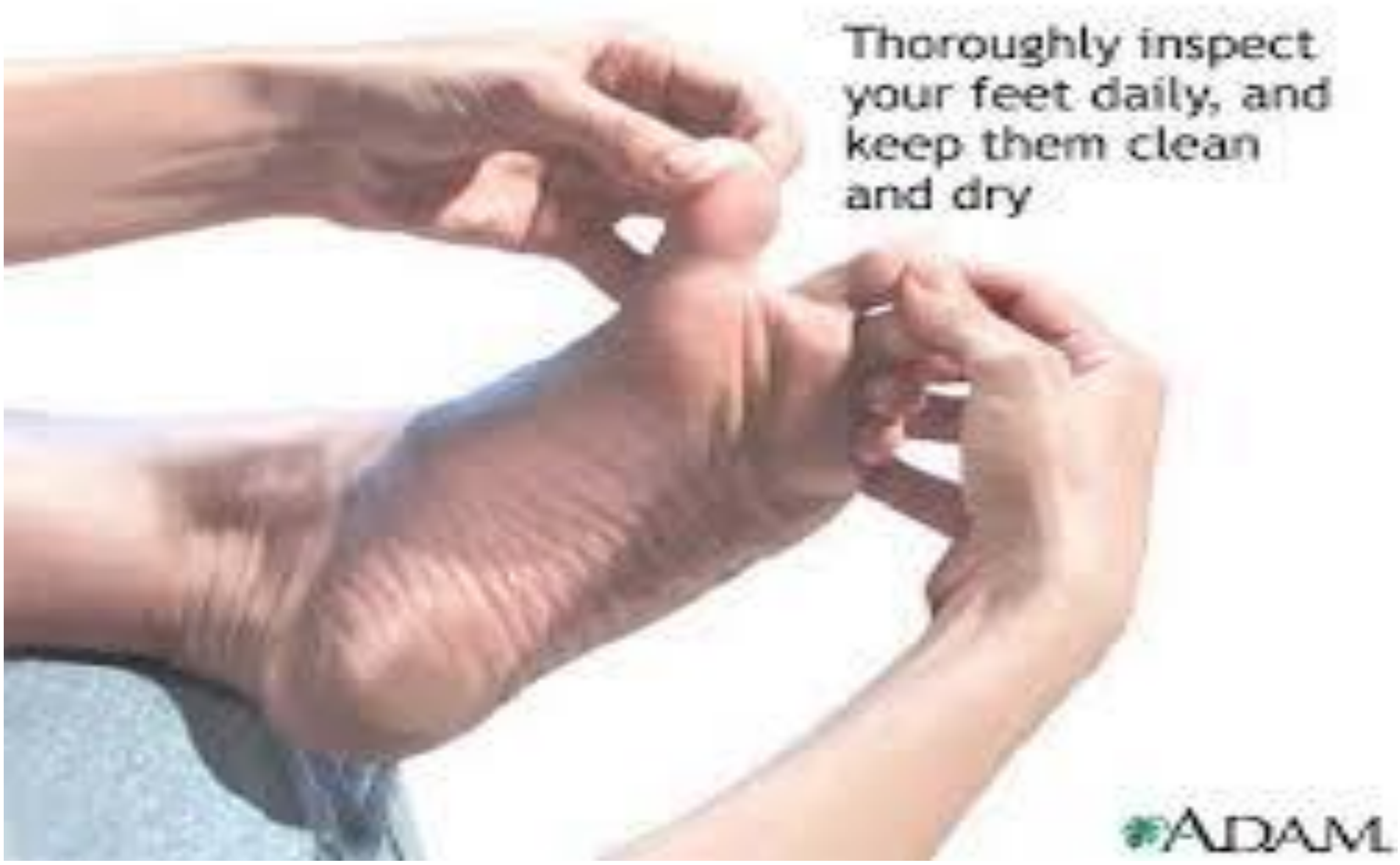
Thoroughly inspect your feet daily, and keep them clean and dry
ADAM.

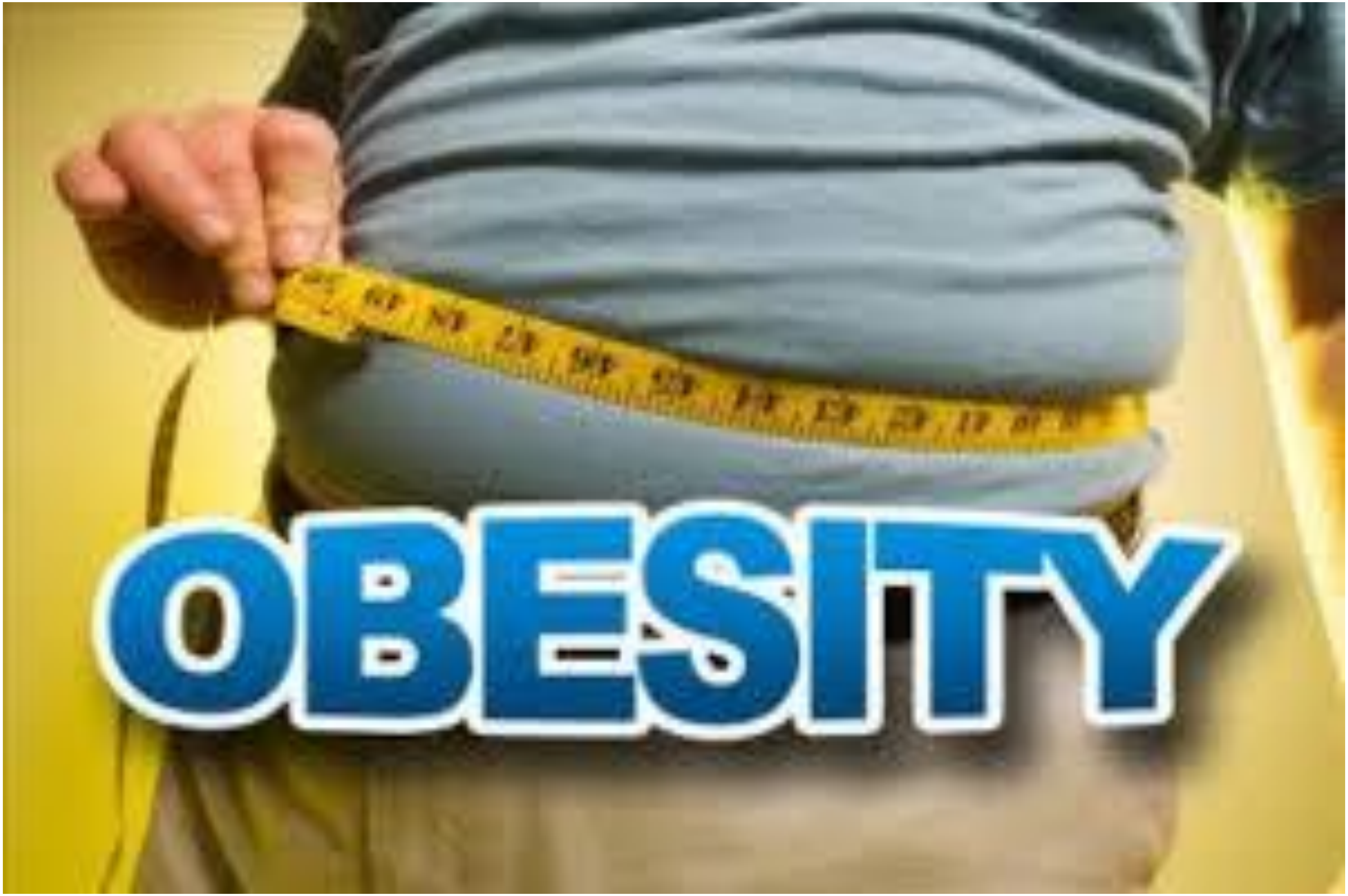
OBESITY

# BMI(Body Mass Index)

Calculate Your

$$BMI = \frac{\text{Weight (kg)}}{\text{Height x Height (metre)}}$$

**BMI Chart**

| | |
|---|---|
| BMI less than 18.50 | Underweight |
| BMI 18.50 - 24.99 | Healthy weight |
| BMI 25.00 - 29.99 | Overweight |
| BMI 30 or more | Obese |

| WHO Classification | Associated risks |
|---|---|
| BMI between 18.5 and 25 : normal weight | Normal |
| BMI between 25 and 30 : overweight | Average |
| BMI between 30 and 40 : obesity | Important |
| BMI above 40 : morbid obesity | Severe |

# Central Obesity

- Waist circumference (WC) recommendation:

✓ Less than 90 cm for men

✓ Less than 80 cm for women

- Even if your BMI is normal, if your WC is high, you are at risk.

# Dyslipidemia/ High blood cholesterol

| Lipid profile | Desirable range |
|---|---|
| Total cholesterol | <200 mg/dl |
| Triglyceride | <150 mg/dl |
| LDL (bad cholesterol) | <100 mg/dl |
| HDL (good cholesterol) | >50 mg/dl |

# Stress

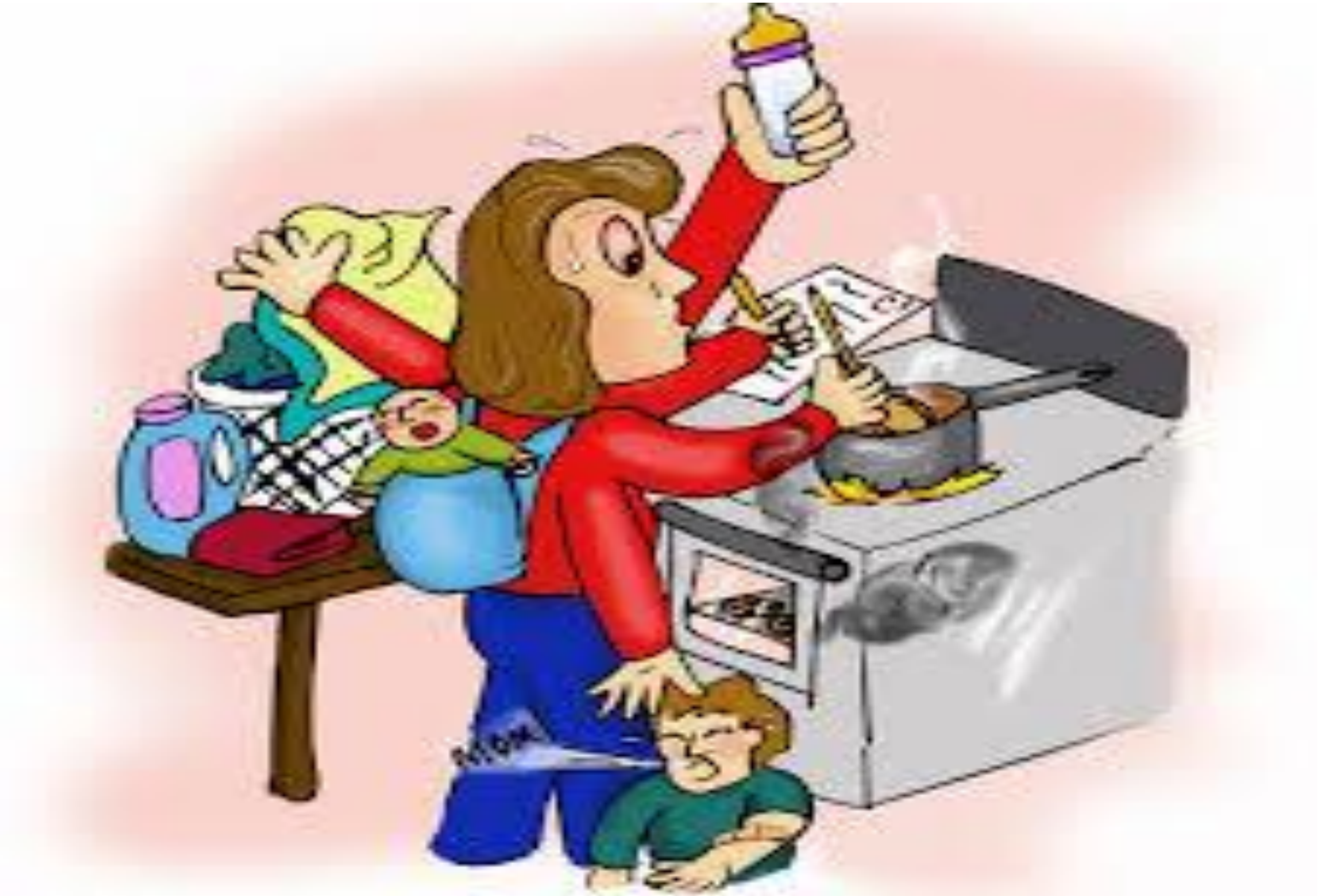

# World Health Day: 7th April
# WHO Theme 2017

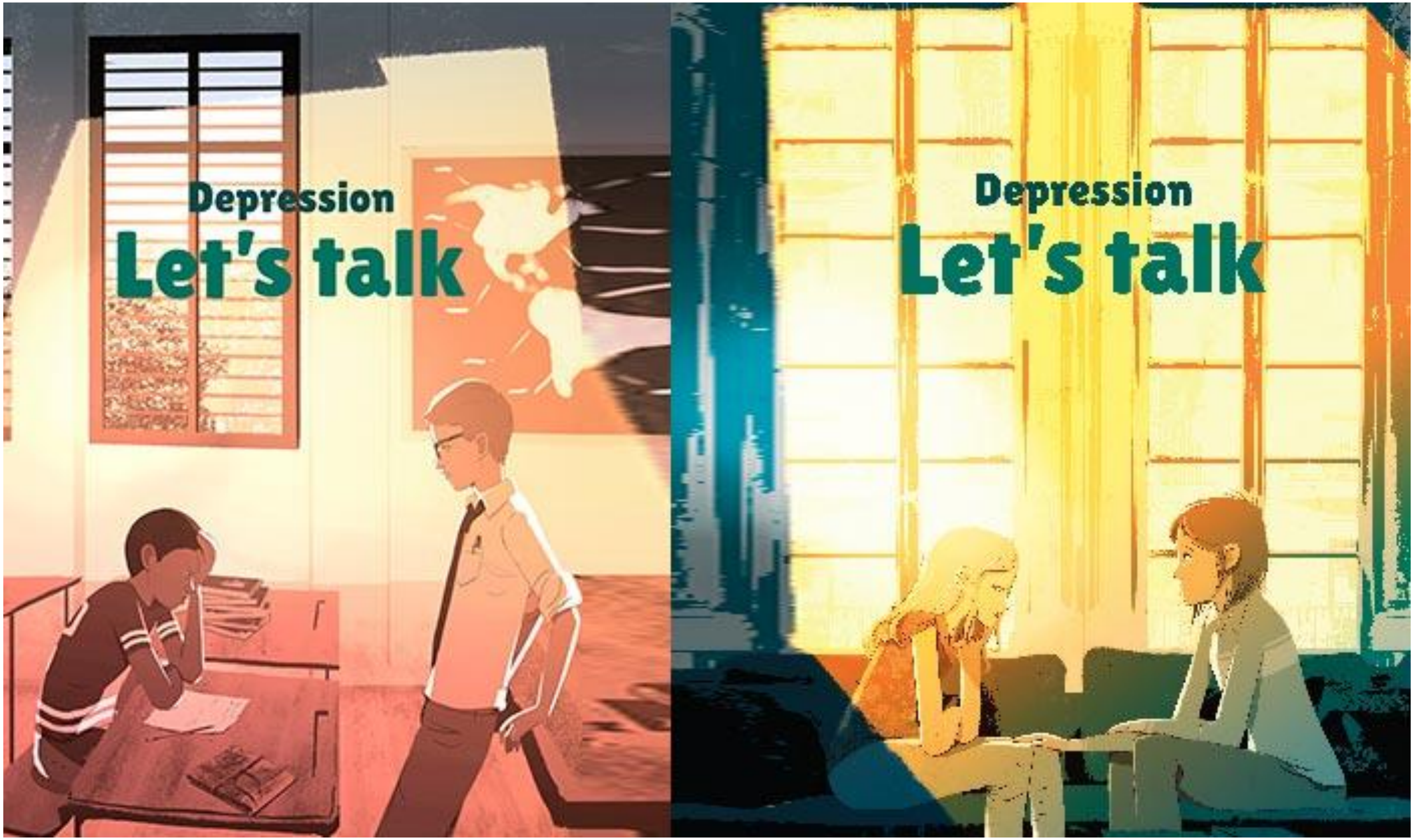

# Tips on Managing Stress

- Positive Attitude
- Support System
- Exercise
- Laughing
- Taking Time for You
- Meditation

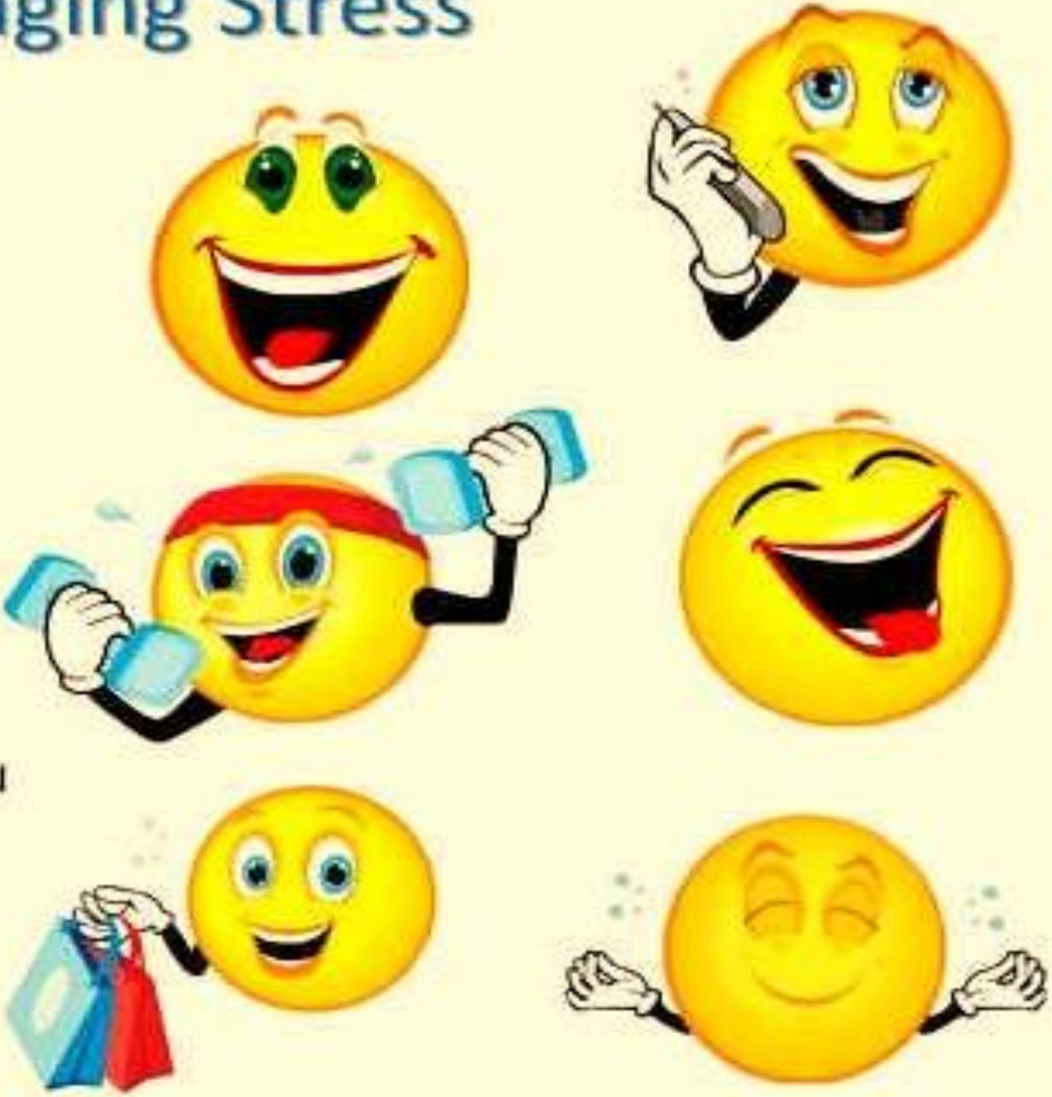

# BENEFITS OF YOGA

**INCREASES**

- FEELING OF WELLNESS
- POSITIVE ATTITUDE
- CONCENTRATION
- FLEXIBILITY
- ENERGY
- SOCIAL SKILLS

**DECREASES**

- ANXIETY/ STRESS
- DEPRESSION
- SUGAR & CHOLESTEROL
- WEIGHT
- BLOOD PRESSURE
- ARTHRITIS / MUSCLE PAIN

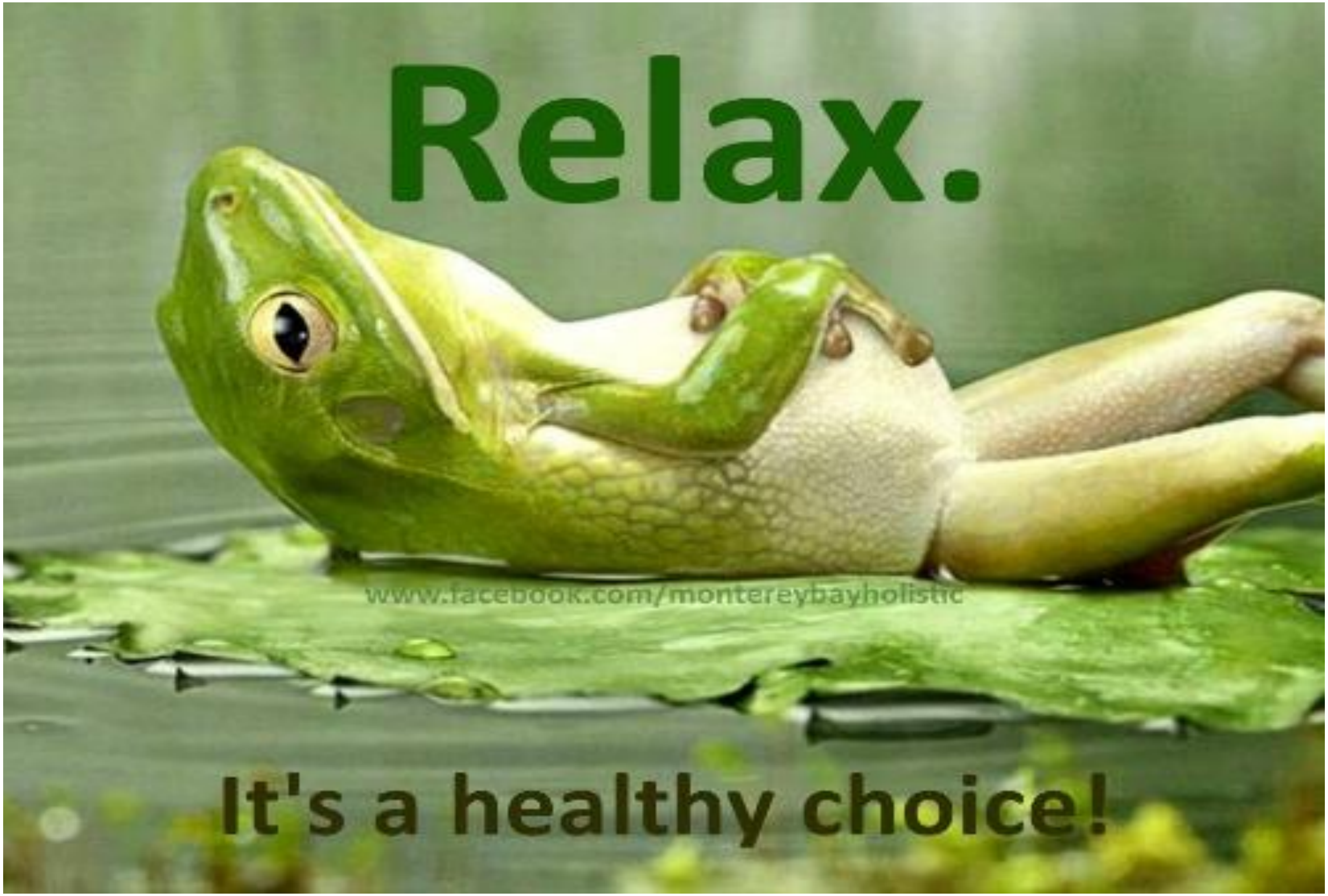
Relax.
www.facebook.com/montereybayholistic
It's a healthy choice!

Do it for life!

# Thank you
