# Supplementary material for: Effect of educational intervention on risk factors of cardiovascular diseases among school teachers: a quasi-experimental study in a suburb of Kolkata, West Bengal, India
Source: BMC Public Health. 2023 Nov 21;23:2304. doi: 10.1186/s12889-023-17227-w (PMC10664257; doi:10.1186/s12889-023-17227-w)
Supplement: Supplementary file 2 — Annexure II: Handout Given to Partcipants along with Intervention [file 12889_2023_17227_MOESM2_ESM.pdf]

Annexure II: Handout Given to Partcipants along with Intervention

Cardiovascular Diseases (CVD): Risk Factors & Prevention

- Most of the risk factors of cardiovascular diseases are **modifiable**. They are-
- Tobacco use
- Harmful use of alcohol
- Physical inactivity
- Unhealthy diet
- Raised blood pressure/ Hypertension
- Raised blood sugar/ Diabetes
- Raised blood cholesterol
- Obesity/ Overweight
- Stress

Tobacco Use

- **Smoking** and using **Smoke Less Tobacco** (e.g.- khaini, gutkha, jarda etc.) **both** increases chance of developing CVD
- "Smokers are more likely to die of lung cancer than CVD"- it is a **FALSE** statement.
- **Q. How to quit?** Ans. Stop **ABRUPTLY.** Do not shift to any other form of addiction. This is the **ONLY** way!

Alcohol Use

- Moderate drinking of alcohol is protective against CVD but **alcohol has lots of other harmful effects on our body**
- If you've had significant alcohol-related problems and are currently not drinking, trying moderate drinking may put you at risk again for alcohol-related problems.
- If you're currently drinking more than the guidelines of moderation, you will reduce your risk for problems by cutting back.

Dietary Recommendations:

- Vegetables including Green Leafy Vegetables: 3-4 servings/day
- Fruits: at least 1 everyday
- Salt < 5 gm / day / person.
- Oils 500 gm / person / month
- Avoid fast food and junk food

Physical activity recommendation:

- Moderate physical activity (e.g.- brisk walking, cycling, swimming, gardening, aerobics, etc.) - at least **30 minutes/ day and 5 days/ week** i.e. **150 minutes/ week**
- Minimum duration of each episode should be **at least 10 minutes**

Hypertension

- Hypertension is blood pressure 140/90 (systolic/diastolic) mm of Hg or higher.
- Self-care: 1) Check BP regularly and 2) Take antihypertensive drugs **regularly** if it is advised by your doctor
- **“If I start taking drugs I have to take lifelong!”-** It is better to start drugs to control BP rather than let hypertension damage your other organs like heart and kidney permanently.

Diabetes

- Normal blood glucose level: FBS < 100 mg/dl and 2 hr PPBS < 140 mg/dl
- Diabetes: FBS ≥126 mg/dl and 2 hr PPBS (on OGTT i.e after 75gm glucose load) ≥ 200 mg/dl
- Diabetes self-care: Diet, physical activity, checking blood sugar regularly, taking drugs regularly, foot care, eye care

Obesity

- Keep your Body Mass Index (BMI) under normal range.
- BMI= Weight in kg/ Height in meter ^{2}

**BMI**	**Classification**
< 18.5	Underweight
18.5- 24.99	Normal
25-29.99	Overweight
≥30	Obese

Central Obesity

- Waist circumference recommendation: **<90 cm for men** and **<80 cm for women**

Dyslipidemia/ high blood cholesterol

**Lipid profile**	**Desirable range**
Total cholesterol	<200 mg/dl
Triglyceride	<150 mg/dl
LDL (bad cholesterol)	<100 mg/dl
HDL (good cholesterol)	>50 mg/dl

Stress

- Tips for managing stress: Stay positive, take support from friends and family, exercise, make time for yourself, engage in hobbies, do meditation and yoga.

Thank you
